# The Local Edge Machine: inference of dynamic models of gene regulation

**DOI:** 10.1186/s13059-016-1076-z

**Published:** 2016-10-19

**Authors:** Kevin A. McGoff, Xin Guo, Anastasia Deckard, Christina M. Kelliher, Adam R. Leman, Lauren J. Francey, John B. Hogenesch, Steven B. Haase, John L. Harer

**Affiliations:** 1 Department of Mathematics and Statistics, UNC Charlotte, 9201 University City Blvd., Charlotte, 28269 NC USA; 2 Department of Applied Mathematics, The Hong Kong Polytechnic University, Hong Kong, China; 3 Department of Mathematics, Duke University, Durham, NC USA; 4 Department of Biology, Duke University, Durham, NC USA; 5 Department of Molecular and Cellular Physiology, University of Cincinnati, Cincinnati, OH USA

**Keywords:** Gene regulatory networks, Inference, Time series

## Abstract

We present a novel approach, the Local Edge Machine, for the inference of regulatory interactions directly from time-series gene expression data. We demonstrate its performance, robustness, and scalability on in silico datasets with varying behaviors, sizes, and degrees of complexity. Moreover, we demonstrate its ability to incorporate biological prior information and make informative predictions on a well-characterized in vivo system using data from budding yeast that have been synchronized in the cell cycle. Finally, we use an atlas of transcription data in a mammalian circadian system to illustrate how the method can be used for discovery in the context of large complex networks.

**Electronic supplementary material:**

The online version of this article (doi:10.1186/s13059-016-1076-z) contains supplementary material, which is available to authorized users.

## Background

Temporally dynamic gene expression programs have been observed in a wide variety of organisms. In some instances, it is believed that the observed temporal dynamics are an emergent property of underlying transcription networks, which consist of interacting collections of transcription factors (TFs) [1–3]. Although it is difficult to assay such transcription networks directly, high-throughput technologies allow the measurement of transcription levels in time-course experiments [4, 5]. However, using such time-course transcriptome data to infer the structure of transcription networks is considered a major problem in computational biology [6, 7]. To date, many inference methods have been proposed for reconstructing gene regulatory networks [8, 9], but successful network inference directly from time-series datasets has remained elusive [8]. In fact, practicing systems biologists continue to rely on the manual curation of network models [2, 3, 10–12]. Indeed, the network inference problem persists in systems biology, despite an abundance of regulatory evidence in the form of TF binding experiments, genetic screens for candidate nodes, and mutant expression profiling experiments.

We are particularly interested in the *functional* components of networks (rather than the most expansive or inclusive network), where the function of the network is manifested by the *dynamics* of the network. By functional network, we mean a network such that an experimental perturbation will likely alter the dynamical phenotype of the network. One of the best examples of a large functional network is the mammalian circadian oscillator, for which the current core network contains about 30 nodes.

Previous methods for network inference from dynamics data may be broadly classified according to the tools involved. Many methods rely on linear statistical models called vector auto-regressive models, including methods based on Granger causality [13–15]. Other popular approaches employ sparse linear regression and related techniques [16–18], calculations of mutual information [19], or dynamic Bayesian networks [20–23]. Most recently, several studies have developed inference methods based on nonlinear ordinary differential equations (ODEs) for the chemical kinetics and a Bayesian formalism on the network structure [24–27]. This article fits into the latter class and extends some of those ideas as follows.

**Fig. 1:**
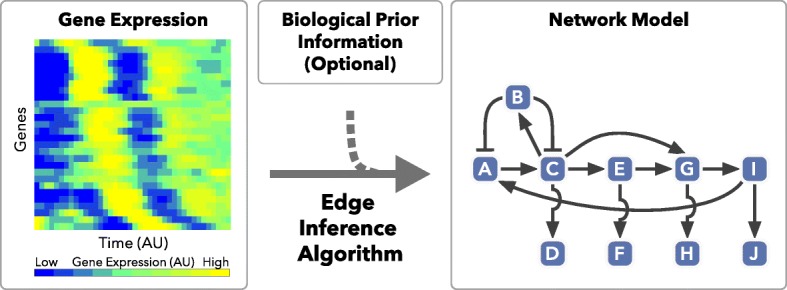
Discovering underlying transcriptional networks from time-series gene expression data. The LEM inference method utilizes time-series gene expression data (*left*) to estimate parsimonious network structures matching the observed dynamics (*right*). In general, LEM will test every input node as a possible regulator of all others. These LEM outputs may be refined by biological prior information (*center*) within a Bayesian framework to generate plausible network models (*right*). *AU* arbitrary units, *LEM* Local Edge Machine

Beginning with time-series gene expression data, the Local Edge Machine (LEM) seeks to find functional network models capable of generating the dynamic behavior of the data (Fig. 1). This approach begins with nonlinear kinetic equations, which provide realistic models of transcription and facilitate interpretability of the resulting models. Furthermore, LEM operates in a Bayesian framework, which accounts for uncertainty, prior information, and robustness in the parameter space. It uses a local approximation to the system of differential equations that relies on sparse priors, which localizes uncertainty and renders the algorithm scalable to complex networks. One interesting feature of our approach is that it provides a coherent framework for modeling both the local motifs (e.g., edges) within the network and the global dynamical behavior of the system. Indeed, using the locally inferred network structure and parameters, LEM produces a complete system of ODEs capable of generating dynamic predictions. Additionally, our approach differs from previous methods in its reliance on the equivalent formulation of ODEs as integral equations, which improves robustness to noise, and in its use of a Laplace approximation of the posterior, which reduces the computational cost by eliminating the need for any Markov chain Monte Carlo (MCMC). In validation studies on both in silico and in vivo data, our method outperforms previously reported methods. We anticipate that this method will be used as a tool in network or pathway discovery settings in which high-fidelity time-course data are available. As the method appears to make informative predictions, we view it as providing a substantial reduction of the hypothesis space that an experimentalist must search [28].

The computational task of inferring network connections from steady-state TF perturbation experiments (gene knockouts or overexpression) has been attempted [8]; however, it is difficult to infer causality, directionality, and the function of network edges from single-point measurements. LEM attempts to overcome these challenges by basing edge predictions on dynamics data. Importantly, though, the abundance of data from perturbation experiments and other regulatory evidence from a given model organism could be used throughout the process of LEM network inference in several ways. Indeed, it may be used to inform the selection of nodes chosen to run through LEM, to inform the structure of the prior information used by the algorithm, and to evaluate the output of the algorithm. In particular, the LEM framework allows for the incorporation of a wide variety of evidence in the form of prior information, including genetic evidence (e.g., gene expression changes in TF targets upon TF knockout or overexpression), physical interaction evidence (e.g., high-throughput genomics experiments, such as ChIP techniques, and database compilation, such as ENCODE [29]), and direct regulation evidence (e.g., the fast-on technique to identify direct TF targets [30]). In our yeast cell-cycle analysis, we include TF function (activator, repressor, or unknown; see Additional files 1 and 2) as prior information to improve LEM inference further. Additionally, we use the available regulatory evidence from various TF binding and genetics experiments (see Additional file 3) to evaluate LEM predictions. We view the development and testing of specific prior distributions based on current regulation evidence as an important direction for future work.

## Methods

### Description of the method

Given a set of genes deemed to be potentially important for network function, LEM takes a Bayesian approach to answer the following question: of all possible regulators, which regulator and regulatory logic (activation or repression) best models the expression dynamics of each gene? Here, we provide a brief description of how the LEM algorithm models the gene expression of each node and scores each possible regulation in the network. For a complete description of the mathematical and computational details, see Additional file 1: Sections 1–4.

Consider a gene regulatory network with a set of *N* nodes, $\mathcal {N} = \{X_{1}, \dots, X_{N}\}$. For *i*=1,…,*N*, we let *X*_*i*_(*t*) denote the expression level of gene *X*_*i*_ at time *t*. The data, denoted by *D*, consist of the observed expression levels of the *N* nodes at *T* time points, $\{t_{j}\}_{j=1}^{T}$.

According to our model, the data are generated according to a system of ODEs, possibly observed with noise. More specifically, for the target *X*_*i*_, our model is that *X*_*i*_ satisfies

1 $$  \frac{\mathrm{d}X_{i}}{\mathrm{d}t} = \alpha_{i} f_{i}(\mathbf{X}(t)) - \beta_{i} X_{i}(t) + \gamma_{i},  $$

where **X**(*t*)=(*X*_1_(*t*),…,*X*_*N*_(*t*)), the function $f_{i} : \mathbb {R}^{N} \to \mathbb {R}$ governs the type of regulation that *X*_*i*_ experiences, *α*_*i*_>0 represents the strength of the regulation, *β*_*i*_≥0 represents the rate of degradation of *X*_*i*_, and *γ*_*i*_≥0 represents the basal rate of production of *X*_*i*_. In general, stochastic effects play a significant role in the dynamics of any individual cell, and such considerations lead one to stochastic differential equations. However, we consider data generated by averaging expression levels over many (∼10^8^) individual cells, and we, therefore, assume that the stochastic effects are insignificant, leading to our use of ODEs.

We use Hill function kinetics to model activation and repression of the target node. Equations of this type are not intended to model each individual aspect of regulation explicitly. Rather, they are intended to subsume multiple levels of regulation (e.g., translation, transcription, chromatin modification, direct binding, etc.) into a single equation with relatively few parameters. In general, one expects biological networks to be sparse [31, 32], and even in cases where this assumption is broken, we seek to identify the most dominant components of a regulation in a given experimental condition. Thus, we consider regulatory functions *f*_*i*_ of the following forms, which correspond to regulation by a single gene:

2 $$  f_{i}(\mathbf{X}) = \left\{ \begin{array}{ll} \frac{X_{j}^{n_{i}}}{{K_{i}}^{n_{i}}+X_{j}^{n_{i}}} & (\text{activation by}~X_{j}), \\ \frac{K_{i}^{n_{i}}}{{K_{i}}^{n_{i}}+X_{j}^{n_{i}}} & (\text{repression by}~X_{j}). \end{array} \right.  $$

More complex regulatory functions *f*_*i*_ could be allowed in the model class if the goal is to infer simultaneous regulation by multiple genes. However, we choose to restrict attention to single regulation, since the information content of time-series datasets at present appears not to support the substantial increase in complexity of the model class that would result from inclusion of combinatorial regulation.

Thus, to specify a system of ODEs completely, as in Eq. 1, for each node *X*_*i*_, one must select a regulator *X*_*j*_, a type of regulation (activation or repression), and a vector of real-valued parameters (*α*_*i*_,*n*_*i*_,*K*_*i*_,*β*_*i*_,*γ*_*i*_). We refer to triples of the form (*X*_*i*_,*X*_*j*_,*a*) or (*X*_*i*_,*X*_*j*_,*r*) as edges, where we interpret (*X*_*i*_,*X*_*j*_,*a*) as the relationship that *X*_*i*_ is activated by *X*_*j*_ and (*X*_*i*_,*X*_*j*_,*r*) denotes that *X*_*i*_ is repressed by *X*_*j*_. Note that these edges are both signed and directed.

The LEM inference method first involves making a local approximation, which allows us to infer the regulation of each node separately, rather than all at once (see Additional file 1: Section 2). To infer the regulation of the target *X* (here we drop the subscript *i* from the above notation without introducing ambiguity), LEM takes a Bayesian approach that relies on the Gibbs posterior principle [33, 34] and a Laplacian approximation in the computation of the posterior distribution.

In general, if *M* is a model (among several) and *D* is a dataset, then Bayes’s rule yields a posterior probability of *M* given the data *D*:

$$p(M | D) = \frac{p(D | M) \pi(M)}{p(D)} \propto p(D | M) \pi(M). $$

Here *p*(*D*|*M*) is the likelihood of the data *D* given the model *M*, *π* is a probability distribution on the possible models, called the prior distribution, and *p*(*D*) is the likelihood of *D* (averaged over all the possible models). If one interprets the prior distribution as our belief in the veracity of each model prior to generation of the data, then the posterior distribution represents the optimal way to update our beliefs in light of the data. If *M* requires an additional choice of parameter *θ* to be a fully generative model, the posterior distribution may be written as an integral over *θ*:

$$p(M | D) \propto \int p(D | M,\theta) \pi(M,\theta). $$

For LEM, we formulate the edge inference problem in a similar manner. Let *X* be a fixed node and *E* an edge with *X* as the target [i.e., *E*=(*X*,*Y*,*a*) or *E*=(*X*,*Y*,*r*) for some node *Y*]. We would like to view *E* as a model for explaining the behavior of *X* and employ the Bayesian framework above to compute its posterior probability. To do so, we need to specify a prior distribution on the set of possible models, which in our case is the set of possible edges with *X* as the target, and we need a likelihood function. Recall that in our model, each edge requires an additional choice of parameter vector *θ*=(*α*,*β*,*γ*,*n*,*K*) (as in Eqs. 1 and 2) in order to specify fully the corresponding differential equation.

As mentioned in the introduction, the prior distribution may be set by the user, and there are many opportunities for integrating other data types in this manner. However, in our implementation it is set as follows. First, we let *π*(*E*) be the uniform distribution over the possible edges that have *X* as a target. For each edge *E* with *X* as the target, we select a priori bounds on each of the parameters in *θ*_*E*_, resulting in a region *R*_*E*_ (contained in $\mathbb {R}^{5}$) of biologically reasonable parameter values (see Additional file 1: Section 3). Once these bounds are selected, we choose the maximum entropy prior distribution subject to these bounds, which is the least informative prior on *R*_*E*_ and ensures that we do not unnecessarily bias the result. This distribution is

$$\pi(E,\theta) = \frac{1}{s \cdot \text{Vol}(R_{E})}, $$

where *s* is the number of edges with *X* as target and Vol(*R*_*E*_) is the volume of *R*_*E*_.

With the prior distribution set, we now turn attention to the likelihood. In fact, as different experimental protocols could lead to significantly different noise models, each of which is likely to be difficult to determine accurately and precisely, we proceed under the assumption that we do not have access to a likelihood function. In such cases, the Gibbs posterior principle [33, 34] states that the optimal method for updating one’s beliefs in light of the data is to replace the likelihood *p*(*D*|*M*,*θ*) by

$$\exp\left(- \ell(D, E,\theta) \right), $$

where *ℓ*(*D*,*E*,*θ*) is an appropriately chosen loss function. We specify a loss function *ℓ*(*D*,*E*,*θ*) as follows. For a triple (*D*,*E*,*θ*), define the function $F : [t_{1},t_{T}] \to \mathbb {R}$ on the points $\{t_{j}\}_{j=1}^{T}$ by

$$F(t_{j}) = \alpha f(\mathbf{X}(t_{j})) - \beta X(t_{j}) + \gamma, $$

and then extend *F* to the whole interval [*t*_1_,*t*_*T*_] by linearly interpolating between these values. That is, if *t*=*ut*_*j*_+(1−*u*)*t*_*j*+1_ for some *j*<*T* and *u*∈(0,1), then let *F*(*t*)=*uF*(*t*_*j*_)+(1−*u*)*F*(*t*_*j*+1_). Now set

$$\hat{X}(t) = \int_{t_{1}}^{t} F(s)~ \mathrm{d}s, $$

and define the loss *ℓ*(*D*,*E*,*θ*) to be the mean squared error between the observed values $\{X(t_{j})\}_{j=1}^{T}$ and the properly shifted model prediction $\{\hat {X}(t_{j})\}_{j=1}^{T}$:

$$\ell(D,E,\theta) = \min_{c \in \mathbb{R}} \frac{1}{T} \sum\limits_{j=1}^{T} \left(X(t_{j}) - \hat{X}(t_{j}) -c \right)^{2}. $$

This choice of loss function is effectively equivalent to the choice of a Gaussian noise model.

With the prior distribution and the loss function now specified, the (marginal) Gibbs posterior probability [33, 34] of the edge *E* given the data is

3 $$  p(E | D) \propto \int_{R_{E}} \exp\left(- \ell(D,E,\theta) \right) \frac{\mathrm{d}\theta}{s \cdot \text{Vol}(R_{E})}.  $$

As is common in many Bayesian methods, the above integral does not have a closed-form solution. We choose to estimate it using a Laplace approximation [35] (see Additional file 1: Section 3). From this approximation, one can see that LEM explicitly favors networks whose dynamics are more robust to a perturbation in the parameter space. In principle, one could attempt to compute other approximations of this integral, including Monte Carlo approximations. However, we have found that the Laplace approximation is computationally fast and produces sufficiently accurate results for our purposes.

Thus, the core output of LEM is *N* different probability distributions—one for each node in the network (see Additional file 1: Section 3.1). The distribution for node *X* should be interpreted as representing our beliefs about which edge is the dominant regulatory interaction (edge) controlling the expression of *X*. There are multiple ways to obtain a single network from this set of distributions, the simplest of which is to select the most likely edge from each distribution.

## Results

### Validation and testing: in silico and yeast cell-cycle data

**Fig. 2:**
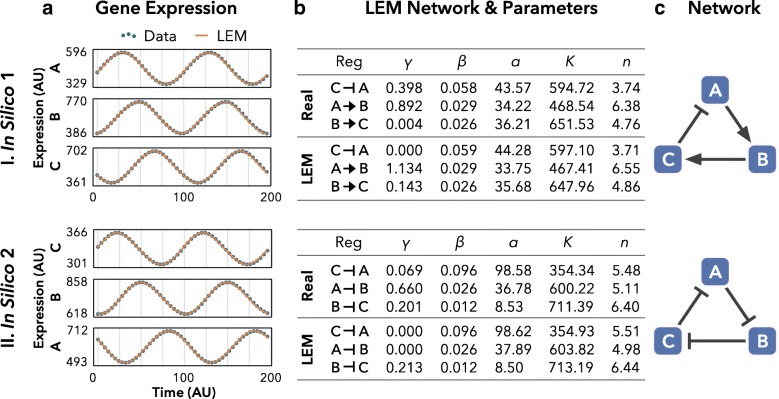
LEM recapitulates network structure and dynamics from time-series data. I, II Oscillatory gene expression data were generated in silico from three-node negative feedback networks (**a**, *dotted lines*). LEM uses ordinary differential equations to simulate the dynamics data for each node (**a**, *solid lines*). The parameters output from LEM closely approximate the parameters used to generate the data (**b**). LEM identifies the most probable regulation on each node, from which the user can generate a composite network diagram (**c**). The top regulations identified by LEM reconstruct the correct network topology for both networks (I in silico 1; II in silico 2). Out of a possible 3^9^=19,683 topologies for three-node networks, LEM detects subtle differences in gene expression curve shapes to identify correctly a three-node negative feedback loop (I) and the repressilator [49] (II). *AU* arbitrary units, *LEM* Local Edge Machine

To begin testing the capabilities of LEM, we constructed synthetic three-node networks that produce oscillatory behaviors under certain parameter sets (Fig. 2 a). We used LEM to estimate the network structure, as well as a fully parameterized system of differential equations. Here LEM perfectly reconstructs the networks and produces parameter estimates for systems of differential equations that generate essentially the same dynamics as the data (Fig. 2 b, c).

**Fig. 3:**
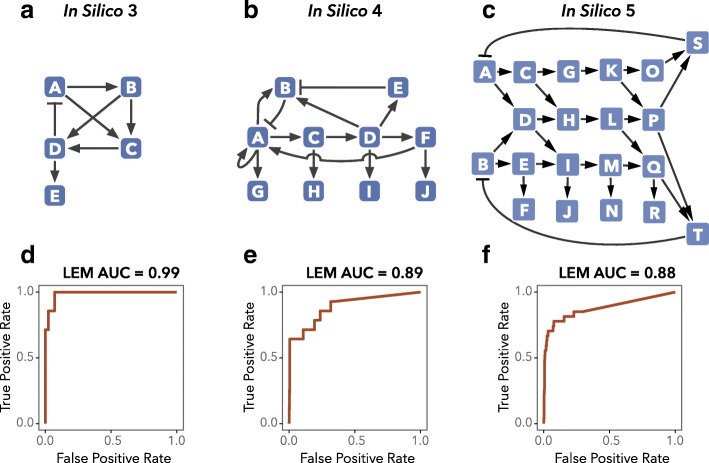
LEM inference performance is robust to increasing network size and complexity. Oscillatory gene expression data were generated in silico for five-, ten-, and 20-node networks (**a** in silico 3; **b** in silico 4; **c** in silico 5, respectively). AUC-ROC scores (**d**, **e**, and **f**) were computed as described in Additional file 1: Section 5; recall that a random ranking of edges should produce an expected AUC-ROC score of 0.5. *AUC* area under the curve, *LEM* Local Edge Machine, *ROC* receiver-operating characteristic

We investigated the scalability of LEM on functional networks by creating networks with oscillating dynamics consisting of five, ten, and 20 nodes, in which regulation of several nodes follows complex logical rules, e.g., AND gates and OR gates [36, 37] (Fig. 3). Note that these networks include a considerable model mismatch, in the sense that complex regulation of individual nodes appears throughout these networks, despite that LEM does not directly infer such regulation. See Additional file 1: Section 9 for details of the construction and parameterization of these networks. As seen in the receiver-operating characteristic (ROC) plots for these examples, LEM’s performance remains strong as the networks scale in size and complexity [see Additional file 4 for sample LEM ROC curves and Additional files 5 and 6 for LEM area under the curve (AUC) ROC and area under the precision-recall curve (AUPR) scores on all in silico networks used for testing].

Comparison of network inference algorithms is itself a difficult task, as both inputs and outputs of various algorithms typically differ in format. Nonetheless, Table 1 depicts the results of comparisons between LEM and several other algorithms designed to handle time-series data (see Additional file 1: Section 5 for details on how these algorithms were compared). Four of the algorithms (TD-ARACNE [19], Inferelator [17, 18], Banjo [20, 21], and Granger Causality [14]), representing distinct approaches to inference, were selected for comparison due to their (or their predecessors’) strong performances in previous testing, including in the DREAM network inference challenges [8]. Additionally, two more recent methods, here called Hill-DBN [22] and Jump3 [25], were selected for comparison. TD-ARACNE uses the information-theoretic concept of mutual information. Inferelator relies on sparse linear optimization techniques. Banjo and Hill-DBN are dynamic Bayesian network algorithms. Granger Causality involves statistical hypothesis testing, and Jump3 relies on a non-parametric inference procedure based on decision trees.

**Table 1:** LEM outperforms existing network inference algorithms on both in silico and biological data

Network	*#* Nodes	LEM (AUC)	Inferelator (AUC)	Granger Causality (AUC)	Hill-DBN (AUC)	Jump3 (AUC)
In silico 1	3	1.0000	0.9000	0.7000	0.5000	0.9000
In silico 2	3	1.0000	0.5667	0.8111	0.3667	0.7222
In silico 3	5	0.9900	0.7857	0.7791	0.4003	0.6794
In silico 4	10	0.8884	0.5541	0.5949	0.5131	0.7727
In silico 5	20	0.8781	0.6789	0.7441	0.6770	0.7540
Yeast cell-cycle 1	17	0.8693	0.6705	0.6893	0.6253	0.6481
Network	*#* Nodes	LEM (MCC)	TD-ARACNE (MCC)	Banjo DBN (MCC)		
In silico 1	3	1.0000	0.0000	−0.5000		
In silico 2	3	1.0000	0.0000	−0.5000		
In silico 3	5	0.7379	0.4528	−0.0624		
In silico 4	10	0.7463	0.0636	0.0294		
In silico 5	20	0.5908	0.2147	0.0086		
Yeast cell-cycle 1	17	0.0478	0.0292	−0.0380		

**Fig. 4:**
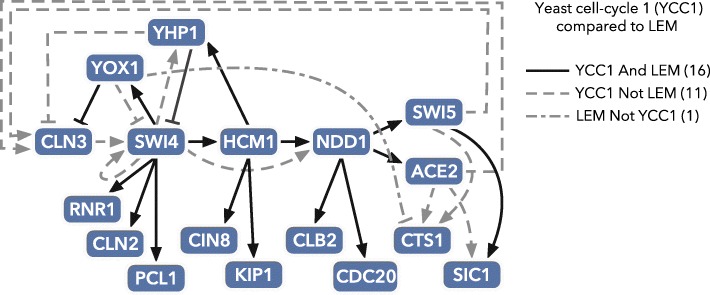
Using data from the budding yeast *Saccharomyces cerevisiae* and biological priors, LEM identifies a core cell-cycle transcription factor network. The topology of the yeast cell-cycle network was manually curated using literature evidence (see Additional file 1: Section 11). We ran LEM with biological prior information (see Additional file 1: Table 2), and we considered an edge to be output by LEM if had a posterior probability of at least 0.4. Edges present in both the cell-cycle network and the LEM output appear in *bold*. Edges that appear in the cell-cycle network but not in the LEM output are *dashed*. The edge that appears in the LEM output but not in the cell-cycle network is *dash-dotted*. *LEM* Local Edge Machine, *YCC* yeast cell cycle

**Fig. 5:**
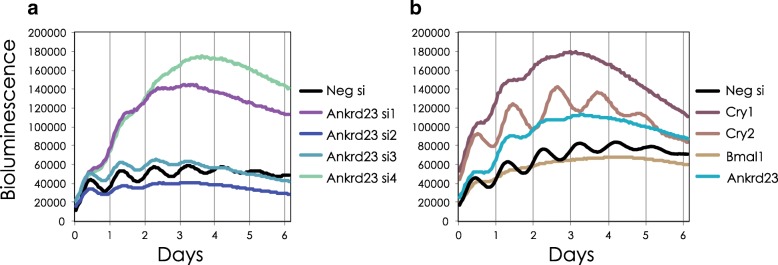
*Ankrd23* knockdown leads to arrhythmicity of circadian bioluminescence output in mammalian clock cells. **a**, **b** NIH3T3 cells stably expressing the mouse *Per2* promoter driven destabilized luciferase reporter gene were reverse transfected with targeted siRNAs against *Ankrd23* (*n*=4), negative siRNA, or pooled siRNAs (*n*=4) targeting known clock genes as controls. Transfected cells were synchronized with forskolin 24 h post-transfection. Bioluminescence was monitored in real time at a 1-h sampling resolution over 6 days. The mean bioluminescence data plotted (*n*=3) is a representative of three independent experiments. **a** Three out of four targeted siRNAs against *Ankrd23* lead to an arrhythmic clock phenotype. **bAnkrd23** siRNA pool (*n*=4) leads to a short/arrhythmic clock phenotype with an increased baseline similar to the Cry1 knockdown control phenotype

Using in silico networks 1–2 (Fig. 2) and 3–5 (Fig. 3), as well as a yeast cell-cycle network (Fig. 4), we compared LEM performance to existing algorithms. AUC-ROC scores labeled (AUC) were used to compare the performance of LEM to Inferelator, Granger Causality, Hill-DBN and Jump3. Matthew’s correlation coefficient (MCC) was used to compare LEM to TD-ARACNE and BANJO, which are binary classifiers and do not output numerical scores for network edges. No biological prior information was used for this comparison. Using dynamics data from each network, LEM better approximates the underlying network model than the other algorithms. See Additional file 1: Section 5 for a complete explanation of AUC-ROC and MCC scoring

*AUC* area under the curve, *LEM* Local Edge Machine, *MCC* Matthew’s correlation coefficient, *ROC* receiver-operating characteristic

To compare these algorithms, we first used several of our benchmark datasets of oscillatory dynamics from in silico networks. Then we examined the performance of the algorithms on transcriptome data generated from time-series experiments on synchronized yeast cells [2] (see Additional file 1: Section 10 for a description of the data and Additional file 1: Section 11 for a description of the curation of a yeast cell-cycle network). As LEM makes more detailed predictions than these algorithms, we weakened its predictive power to make these comparisons. Nonetheless, as shown in Table 1, LEM outperforms these algorithms on both in silico and in vivo networks.

To demonstrate the performance of LEM on biological data, we begin with time-series data collected in the study of the transcriptional oscillator underlying the yeast cell cycle [2]. Based on these data, as well as on previously available data, a tentative network model was previously manually curated [2]. We created a network consisting of the previously published network [2] and some other known targets (see Additional file 7 for the list of genes, Additional file 1: Section 11 for a discussion, and Additional files 3 and 8 for almost 100 citations supporting this network). Taking this network as the gold standard, we found that the LEM predictions obtained an AUC-ROC score of 0.8693, indicating that the LEM predictions were highly informative with respect to the manually curated network. Indeed, as can be seen in Additional file 9, the gold standard edges are ranked highly by LEM, even without the inclusion of any prior information. Recognizing that all model networks represent an approximation of the underlying reality and are subject to revision, we also constructed both smaller (more restrictive) and larger (more inclusive) networks from the available data (see Additional files 10, 11, and 12 for the networks, Additional file 1: Section 11 for a discussion, and Additional files 3 and 8 for citations). We then compared the output of LEM to these networks, as seen in Additional file 5.

Since LEM takes a Bayesian approach, it also easily incorporates prior information. For the testing on the yeast cell-cycle data, we used a simple form of prior information: each node should appear exclusively as an activator or as a repressor. For example, if TF *Y* is known to be an activator (repressor) of its target genes, then inclusion of this function as prior information would exclude any regulation of the form *X* is repressed (activated) by *Y*. Note that this type of restriction appears only according to the user-defined prior information, and by default a TF is allowed to appear as both a repressor and an activator (which appears to be the case in many systems, especially in mammals [38–41]). To get an idea about the performance of LEM with this type of prior information, we first simulated this type of prior information with our benchmark in silico datasets (see Additional file 1: Section 6 for details and Additional file 13 for results).

For the yeast cell-cycle networks, we obtained this prior information from experimental evidence previously reported in the literature (most of which may be found in YEASTRACT [42] or SGD [43]); see Additional file 2. Inclusion of this prior information yields substantial improvement in the inference (see Additional files 5 and 14). In particular, in the presence of prior information about the possible roles played by each node (i.e., activator or repressor), LEM predictions obtained an AUC-ROC score of 0.9889 and produced the network drawn in Fig. 4. Furthermore, partial inclusion of this information incrementally improves the results (see Additional file 1: Section 6 and Additional file 15), indicating that LEM is a useful tool for biologists who only partially understand the function of important nodes in their network of interest. Core TFs in the cell-cycle network are under complex regulation, which is not always captured by LEM. However, LEM correctly identifies many outputs from cell-cycle TFs. Thus, given a TF of interest in a gene regulatory network, LEM is a useful tool for scoring probable targets of that TF based on time-series gene expression data.

### Validation and testing: mammalian circadian data

Next, we used LEM to discover new nodes in a complex incomplete biological network. The mammalian circadian clock is a transcriptional network that regulates gene expression in tune with the 24-hour light/dark cycle. While genetics and biochemistry have identified many core components/nodes of the circadian network, its full complement of nodes and its topology remain uncharacterized. Recently, Zhang and colleagues built an atlas of circadian transcription from 12 mouse organs and found that at least 43 % of the protein-coding genes are under clock control [44]. In this and other data, known clock genes tend to have the highest amplitude and most statistically significant rhythms [45]. Reasoning that new clock genes are likely to have similar dynamics, we used LEM to search these data for new clock genes.

First, we assembled a list of 31 high-confidence core circadian clock genes (see Additional file 16). Next, we used a suite of periodicity detection algorithms (see Additional file 1: Section 12) to find clock-regulated genes in each of the 12 mouse organs. Reasoning that novel circadian regulators are likely to regulate the known circadian core components, we ran LEM to estimate the probability that each candidate regulates a known clock gene. By summing these probabilities across all known circadian core components in all 12 organs, we calculated a score reflecting how likely each periodic gene is to regulate known core components. We selected a threshold of 0.1 for significance of this score, identifying 333 potential regulators. Notably, ten known clock genes were in this list.

Clock genes regulate each other. To winnow down this list, we used the liver data and found 205 candidates that were regulated by known clock components (see Additional file 17). We focused on the liver, as it is the organ with the strongest regulated circadian rhythms and best companion datasets (e.g., ChIP-seq data on known clock components). Known clock genes are TFs, kinases, and ubiquitin ligases. Reasoning that new components are likely to be in these classes as well, we filtered the list of 205 genes down to 34 genes in these or other plausible classes. Based on practical considerations (time and cost), we chose to conduct functional studies on the ten highest ranked genes from this list of 34. We consider the other 24 genes to be good candidates for future experimental work.

We used NIH 3T3 fibroblasts with an integrated PER2:Luc reporter, RNAi, and kinetic luminescence imaging to test the effect of knockdown of each of these components on clock function in vitro. *Rnf152* and *Ppp1r3c* were not expressed in NIH 3T3 cells, so we substituted *Nup62* (a nuclear pore complex factor) and *Fus* (a DNA/RNA binding protein). Five out of these ten knockdowns led to significant changes in circadian period or loss of circadian rhythms (see Additional file 18 for results), and four of these five remain significant after multiple hypothesis correction (see Additional file 19), indicating their requirement for normal circadian rhythms in 3T3 cells. An earlier whole-genome screen in a cell model found knockdown of ∼2.5 % of genes had circadian rhythm phenotypes including shortened or lengthened period and arrhythmicity [46], suggesting LEM provided a significant enrichment over background (Fisher’s exact test gives a *p* value of 1.089×10^−5^ after multiple hypothesis correction). Interestingly, *Ankrd23*, a poorly characterized globular protein, was found to be critical for circadian rhythmicity, as *Ankrd23* knockdowns were arrhythmic (see Fig. 5), the most profound phenotype observed. Taken together, these results show how LEM can be combined with biological information and functional validation to find causal nodes in complex biological networks.

### Further validation and testing: noise, partial information, and computational resources

In addition to the studies described above, we used in silico networks to test the performance of LEM with respect to changes in several other qualities of the data: noise [47], incomplete prior information, and non-periodic systems. To test for robustness against noise, we added truncated Gaussian noise to the data and computed the corresponding AUC-ROC score for LEM. Our tests covered a range of noise scales, where the largest noise scale was chosen so that the variance of the noise was 32 % of the variance of the signal. For a full description of our noise testing and precise results, see Additional file 1: Section 6 and Additional file 19. Based on these experiments, it appears that noise of this type does not greatly reduce the performance of LEM.

We also examined how the performance of LEM changed with the inclusion of prior information. To mimic the type of prior information that we used in the yeast cell-cycle analysis, each node in our in silico networks was assigned an identity, either repressor, activator, or both. We incorporated this information in our prior distribution as follows: if *Y* is an activator, then all edges in which *Y* appears as a repressor have prior probability 0; if *Y* is a repressor, then all edges in which *Y* appears as an activator have prior probability 0; and if *Y* is both, then no change is made to the prior distribution. Next we tested the performance of LEM under increasing access to this prior information. For increasing numbers of nodes, we randomly selected nodes and included their identities as prior information, computing LEM’s AUC-ROC score in each case. As mentioned previously, partial inclusion of this information incrementally improves the results (see Additional file 1: Section 6 and Additional file 15).

Since we initially focused on producing in silico datasets with periodic behavior, we also asked whether LEM could infer network structure and parameters from non-periodic data. For this testing, we generated several non-periodic benchmark datasets in silico, and we used AUC-ROC as a measure of LEM’s performance. LEM appears to perform as well on these data as it does on oscillating data from networks with the same number of nodes (see Additional file 1: Section 7 for results and Additional file 5 for a comparison), with AUC-ROC scores between 0.8 and 1.

Lastly, we evaluated the computational requirements imposed by the LEM algorithm. These requirements can be large, since they depend quadratically on the number of nodes under consideration (see Additional file 1: Section 4 for a precise description of LEM’s implementation and computational complexity, including run-time tables). However, since LEM involves separately computing an approximate posterior distribution on the possible regulations of each node, it is highly parallelizable. Leveraging parallel computations, we observed that LEM is scalable to large networks of the order of hundreds of nodes.

## Discussion and conclusions

We have presented LEM as a tool to prioritize hypotheses for gene regulatory network structures. After validating the approach on in silico networks, we first compared LEM outputs to a gold standard gene regulatory network established by physical evidence gathered from ChIP-on-chip studies in a well-characterized budding yeast cell-cycle transcription network. In this analysis, outputs of LEM, which we view as functional edges, consisted of many of the edges previously characterized, along with one novel edge (YOX1 repressing CTS1), which provides an example of a potential discovery. LEM does not identify all edges that were detected by ChIP studies, and there are several possible explanations of this. For one, LEM is designed to identify the dominant regulatory signal in a given experimental condition, and therefore, it is possible that the gold standard edges not found by LEM are of secondary importance. Furthermore, we speculate that physical binding does not always predict a functional relationship between regulator and target in the conditions that were observed experimentally.

If we relax the *p*-value cutoffs used to construct the gold standard network, we obtain a more complex network with additional nodes and edges that have less experimental support (the yeast cell-cycle 5 network, see Additional file 12 and Additional file 1: Section 10). In general, we find that LEM (along with other algorithms) has a harder time finding evidence for this network in the time-series data. As seen in Additional files 5 and 6, all algorithms perform poorly when this network is treated as the gold standard. Indeed, all AUC scores are close to 0.5, which is what one would expect if edges were ranked randomly. This outcome suggests that there is little support in the time-series data for this as the underlying network.

To demonstrate how LEM could be applied to study larger biological networks, we used LEM to predict novel members of the circadian transcription network, for which both the regulators and the topology are incompletely characterized. Using LEM to look for novel regulators that both receive and transmit regulatory edges to known circadian network nodes, we generated a candidate list of about 200 potential circadian regulators, a dramatic reduction from the thousands of circadian oscillating genes that periodicity-detection algorithms reported in Zhang et al. [44]. For evidence that these results are informative, note that four regulators ranked in the top 20 have been previously shown to have circadian function (see Additional file 17). Furthermore, in a preliminary screen, we found that four out of ten tested genes from the LEM list showed a significant circadian phenotype, despite that previous high-throughput screens found that about 2 % of the genome has a circadian phenotype (see Additional file 18). In light of this performance, we believe that LEM is a powerful tool for reducing a hypothesis space while inferring network topology from the available data.

The issue of non-identifiability of network models for gene regulatory networks has been recognized [6, 7] but not widely studied. This issue arises when distinct networks (i.e., network topologies) have the capability of generating the same dynamics within similar parameter regimes. By definition, no inference algorithm can distinguish between such non-identifiable pairs of networks. Since LEM takes a Bayesian approach, it implicitly rewards models that are robust to changes of parameters (see Additional file 1: Section 3 for a theoretical justification and Additional file 20 for examples). Thus, if two distinct models generate the same dynamics, then LEM will place a higher posterior probability on the more robust model. Although LEM cannot overcome the theoretical limits on inference placed by non-identifiability issues, we observe that LEM, nonetheless, performs quite well, as evidenced by its ability to find global systems of differential equations that fit the data (see Additional file 1: Section 8 and Additional file 20 for examples). This phenomenon also appears in yeast cell-cycle network 1, where LEM does not capture all of the gold standard edges, but it does generate dynamics that closely approximate the observed data (see Additional file 21). Predictions made by LEM for yeast cell-cycle network 1 that were not previously identified by experiments are in the process of being tested.

In addition to the theoretical non-identifiability discussed above, there is a practical issue that arises when the data are not informative enough to distinguish between several models. This situation may arise when one considers sparsely sampled or noisy data, and it calls for additional data from other experimental conditions, such as genetic perturbations. If such data become available, they may be integrated into the framework of LEM via prior information.

As the size of a network grows, the degree of non-identifiability may also increase, since many nodes can present similar dynamics. Such an increase in non-identifiability will necessarily limit the performance of any edge inference algorithm on large networks. To illustrate this point, we created two additional in silico datasets, each with 100 nodes (see Additional files 22 and 23 for the network diagrams and Additional files 24 and 25 for the performance of the inference algorithms). By design, the network in silico 23 contains an extreme amount of redundancy, with 97 of the nodes having exactly the same time series. However, this redundancy is concentrated within the network in such a way that only one edge (out of 100) lacks identifiability, leading to very strong performance by LEM and other edge inference algorithms in our ROC analysis. The network in silico 24 has some practical non-identifiability, in the sense that some nodes have very similar time series (although strictly speaking no two nodes are exactly the same). Accordingly, the performance of the inference algorithms suffers slightly on this dataset. Despite that both of these networks have 100 nodes, LEM is able to infer the correct edges with high accuracy. In summary, non-identifiability (not size) appears to be the main factor limiting the accuracy of LEM.

One of the simplifying assumptions in LEM is that each node in the network has only one dominant regulator controlling its expression level, which is in contrast to some other algorithms, such as Inferelator, that allow one to model regulation with some form of combinatorial control. In principle, one could modify LEM to include such combinatorial terms. However, we believe that the data available in the foreseeable future will not be informative enough to overcome the increase in computational and statistical complexity that would be introduced by these terms, and therefore, inclusion of these terms at this time would result in longer run times and more overfitting. Furthermore, our results indicate that the present version of LEM performs well, even when the generating networks are known to contain combinatorial regulation. One possible explanation for this performance is that even when a gene experiences combinatorial regulation, at least one of the regulators fits the target data reasonably well by itself. In such cases, LEM will typically reward that regulator with a high score, leading to strong performance in a ROC analysis.

In Additional file 1: Section 4, we give details about the computational burden of LEM. Other algorithms that combine detailed differential equation models with a Bayesian formalism tend to employ MCMC to approximate the posterior distribution(s) [26, 48]. In general, LEM avoids the need for any MCMC, thereby reducing the computational burden. In an illustrative comparison against the method of Mazur et al. [48] on four small networks with three nodes, LEM runs substantially faster (see Additional file 26). We did not compare LEM directly to CheMA [26], as the available implementation is designed for protein signaling networks mediated by phosphorylation and therefore, it is not applicable in our setting. Some algorithms that rely on different underlying techniques run faster than LEM, such as Granger Causality [14] and Inferelator [17, 18]. Nonetheless, the parallelizability of LEM makes it applicable to large networks, and the improvement in inferential accuracy demonstrated by LEM over previous methods suggests that it is an especially valuable tool in the search for *functional* networks or network components, which are typically moderate in size.

In the examples considered here, we have focused largely on periodic time series of gene expression, as they clearly result from functional networks, but there is nothing inherent to LEM that limits its utility to gene expression. Indeed, we expect it to generalize as well to other dynamic processes, such as signal transduction pathways or developmental networks. In future work, we intend to extend LEM to allow for the explicit modeling of other cellular processes, such as phosphorylation and ubiquitination.

## Additional files

Additional file 1 Supplementary Information. This document contains 13 sections, each of which provides supporting information for some aspect of the study. Additionally, this document contains five internal tables. (PDF 273 kb)

Additional file 2 Table: Biological prior information about gene function used for LEM inference on yeast cell-cycle networks 1–5. LEM can incorporate biological priors to remove any impossible edges (e.g., a TF known to be an activator cannot repress a given target), which improves inference on real data (see Additional files 5 and 13). Here we provide literature evidence for TFs that function as an activator of target gene transcription, repressor, or both. Genes marked N/A do not function as TFs. Note 1. CLN3 is included as a regulator in networks 1, 2, and 5 because it is known to inhibit Whi5 at cell-cycle START [2]. The other cyclins, CLN2 and CLB2, are not included as regulators because their effects on TFs are redundant with other edges included in the networks (instead, CLN2 and CLB2 are used to represent canonical targets of TFs in the networks). Note 2. PLM2 is lacking specific literature evidence for its role as a TF activator. However, PLM2 is a paralog of TOS4 from the *S. cerevisiae* whole-genome duplication, and we propose that the activator function is shared between the paralogs. (XLSX 43 kb)

Additional file 3 Table: Evidence for regulatory interactions in yeast cell-cycle networks 1–5. Each row corresponds to a regulatory interaction (edge), where an upstream regulator acts on a target gene. *p* values from four high-throughput chromatin immunoprecipitation (ChIP) studies are shown to provide evidence (when available) for a regulator transcription factor (TF) binding to a target promoter [11, 50, 51]. ChIP *p* values were combined using Fisher’s method [52]. Combined *p* values less than 0.001 were considered high-confidence evidence for a given edge (shown in bold red). Where available, edges are supported by additional literature references (see Additional file 8). In the absence of ChIP data, literature evidence was used to determine edges. Evidence for many edges provided here is also documented in the YEASTRACT database [42]. The direction of each interaction (activation, repression, or N/A unknown) is derived from the YEASTRACT database, literature evidence, and/or biological priors about gene function (see Additional file 1). (XLSX 75 kb)

Additional file 4 Figure: ROC plots of LEM together with the results of TD-ARACNE and Banjo. For each of the six networks in Table 1 (in silico 1–5 and yeast cell-cycle network 1), we plotted the ROC curve generated by LEM for the signed directed-edge classification problem. Additionally, we plotted the corresponding results for the binary classifiers TD-ARACNE (marked with a triangle) and Banjo (marked with a square). See Additional file 1: Section 5 for details of these comparisons. (PDF 106 kb)

Additional file 5 Table: Comparison of LEM to existing algorithms on both in silico benchmark datasets and in vivo datasets. As described in Additional file 1: Section 5, LEM was compared to Inferelator, Granger Causality, Hill-DBN, and Jump3 using both AUC-ROC scores and AUPR scores and to TD-ARACNE and Banjo using MCC. See Additional file 1: Section 9 for a description of the in silico networks and Additional file 1: Section 11 for a description of the yeast cell-cycle networks. No prior information was used in these comparisons. Replicates 1 and 2 indicate the biological data input to LEM for predictions on yeast cell-cycle networks 1–5. (PDF 44 kb)

Additional file 6 Table: Comparison of LEM to existing algorithms on the unsigned directed-edge classification problem. LEM was also compared to existing algorithms on the unsigned directed-edge classification problem, in which the sign of the edge (activator or repressor) is removed from consideration. (PDF 43 kb)

Additional file 7 Table: *Saccharomyces cerevisiae* gene expression dynamics used in this study. The wild-type gene expression data of 28 genes over approximately two cell cycles were obtained from previous work [2]. Expression values from each profile were smoothed from 13 experimental time points to 40 time points by fitting a cubic spline (the function splinefun in R package stats). A Rescon Ltd. tool was used to rank the splined profiles by 50 % of the peak expression value. Genes are ordered from earliest (top) to latest (bottom) half-maximal expression and presented in the heat map in Fig. 1. (XLSX 51 kb)

Additional file 8 Table: Compilation of literature that supports regulatory interactions in yeast cell-cycle networks 1–5. Numbers in the first column match literature citations from Additional files 2 and 3. Each number corresponds to a different study that provides experimental evidence for a regulatory interaction. ChIP studies provide binding evidence for a TF to a target gene promoter. Genetic studies provide directional evidence for how a TF influences the expression of a target gene. Phosphorylation and other protein–protein interaction studies provide evidence for how kinases regulate TF activity, localization, and/or binding affinity. (XLSX 40 kb)

Additional file 9 Figure: Ranking of all possible regulations in yeast cell-cycle network 1 by LEM. Each column is a list of possible controls for a single target node. The control mechanisms within each column are rank ordered by LEM according to the posterior likelihood (descending likelihood). For example, in the top left, LEM finds that CTS1 is activated by SIC1. Entries in green text correspond to the gold standard edges from yeast cell-cycle network 1. (EPS 1096 kb)

Additional file 10 Figure: Network diagram of yeast cell-cycle 1. Genes were ordered relative to the timing of their peak transcript level during the cell cycle (where CLN3 represents START, and ACE2/SWI5 marks the M-G1 transition). Nodes and edges were selected as described in previous work [2]. High-confidence transcription factor targets (RNR1, CLN2, PCL1, CIN8, KIP1, CLB2, CDC20, CTS1, and SIC1) were added to increase network complexity for testing the capabilities of LEM on real data. Pointed arrows represent activation, and blunted arrows represent repression of target gene expression. All regulatory interactions are supported by literature evidence (see Additional file 3). In this network, SWI4 serves as a proxy node for the SBF and MBF complexes, which activate a large and overlapping program of approximately 200 genes at the G1-S transition. NDD1 serves as a proxy node for the SFF (Swi Five Factor) complex, which activates a later program of gene expression in S-G2/M. Network diagram of yeast cell-cycle 2. Genes were ordered relative to the timing of their peak transcript level during the cell cycle (where CLN3 represents START, and ACE2/SWI5 marks the M-G1 transition). Nodes and edges were selected as described in previous work [2]. Pointed arrows represent activation, and blunted arrows represent repression of target gene expression. All regulatory interactions are supported by literature evidence (see Additional file 3). Proxy genes for complexes are as described for yeast cell-cycle 1. (EPS 315 kb)

Additional file 11 Figure: Network diagram of yeast cell-cycle 3. Genes were ordered relative to the timing of their peak transcript level during the cell cycle. Nodes and edges were selected as described in previous work [3]. Pointed arrows represent activation, and blunted arrows represent repression of target gene expression. All regulatory interactions are supported by literature evidence (see Additional file 3). Proxy genes for complexes are as described in Additional file 10. Network diagram of yeast cell-cycle 4. Genes were ordered relative to the timing of their peak transcript level during the cell cycle. Nodes and edges were selected as described in previous work [3]. Pointed arrows represent activation, and blunted arrows represent repression of target gene expression. All regulatory interactions are supported by literature evidence (see Additional file 3). Proxy genes for complexes and the addition of canonical target genes were applied as described in Additional file 10. (EPS 324 kb)

Additional file 12 Figure: Network diagram of yeast cell-cycle 5. All transcription factor (TF) nodes from [2, 3] and expanded components of TF complexes were included in this network model (with the exception of ASH1, a daughter-specific repressor). Genes were generally ordered by the timing of peak expression and spatially optimized for network visualization. Pointed arrows represent activation, and blunted arrows represent repression of target gene expression. All regulatory interactions are supported by literature evidence (see Additional file 5). (EPS 379 kb)

Additional file 13 Table: AUC-ROC scores for LEM on in silico networks with observational noise and prior information. As described in Additional file 1: Section 6, we added truncated Gaussian noise to the expression of each gene individually. The column labeled “Noise Level” indicates the variance of the noise as a percentage of the variance of the gene expression. We report the AUC-ROC score for LEM for five in silico networks with increasing access to prior information. (PDF 39 kb)

Additional file 14 Table: Comparison of LEM with priors to existing algorithms on in vivo datasets. In this comparison, each algorithm has access to additional prior information, in the form of a known identity for each node (activator, repressor, neither, or both/unknown). See Additional file 1: Section 11 for a description of the yeast cell-cycle networks used. (PDF 35 kb)

Additional file 15 Table: AUC-ROC scores for LEM on yeast cell-cycle networks with partial access to prior information. For each of the yeast cell-cycle networks, we collected the known identities of the nodes: activator, repressor, both/unknown, or neither (see Additional file 2). The number of such pieces of information for each network is presented in the column labeled “# Priors.” The other columns present the average AUC-ROC score obtained by LEM on each network after randomly selecting the indicated fraction of the possible pieces of prior information as input to LEM 100 times (except that no random selections are necessary in the columns denoted “NoPrior” and “FullPrior”). (PDF 30 kb)

Additional file 16 Table: Compilation of literature that supports the designation of circadian core nodes. The first column gives the names of the genes that were considered to be circadian core genes in our analysis. The second column provides any alternative gene names, and the third column lists literature references that support the designation of the corresponding gene as being part of the circadian core. (XLSX 54 kb)

Additional file 17 Table: Ranking of candidate circadian regulators. We used LEM to discover new potential regulators of circadian core nodes (see Additional file 16) by first selecting the top periodic gene sets from 12 mouse organs (see Additional file 1: Section 12) and then using LEM to identify genes that appear likely to regulate core elements in multiple organs. We obtained a final list of 354 candidate genes (333 top potential regulators plus the 21 core nodes that do appear in the set of 333), of which 205 were either in the set of 31 known core genes or passed the JTK_CYCLE periodicity cutoff of 0.1 for the mouse liver dataset. This table lists the 205 gene common names (column 1), mouse genome database IDs (column 6), and microarray probe IDs (column 5). Additionally, the table marks core nodes with an “x” (column 7) and provides the JTK_CYCLE periodicity *p* value (column 11). Next, we ran full LEM with 205 nodes using mouse liver data. For each of the 205 candidates, we extracted the maximum LEM probability score for any core element targeting each candidate (column 2) and the maximum score for each candidate regulating any core node (column 3). The candidate list is ranked by the product of these two probabilities (column 4). Finally, we compared our candidate list to a compilation of genes with known circadian function (column 7), reviewed by Zhang et al. [12]. (TXT 20 kb)

Additional file 18 Table: Circadian phenotypes of target genes screened by RNAi in NIH3T3 Per2:Luc circadian bioluminescence reporter cells. The *p* values for cycling were calculated using a wavelet-based method [53]. The significance of period length changes was determined using a two-way *t*-test. “AR” is an abbreviation of “arrhythmic.” A *p*-value cutoff of 0.05 was used to determine whether the period length change from Neg si treatment was statistically significant. Five out of ten target genes were found to have a significant circadian phenotype. (PDF 36 kb)

Additional file 19 Table: Multiple hypothesis corrections for analysis of circadian target genes. The column “BHq val” contains the Benjamini–Hochberg corrected *q* values [54]. Four out of ten candidate genes remain significant after multiple hypothesis correction. (PDF 30 kb)

Additional file 20 Figure: Comparison of ODE systems inferred by LEM to networks in silico 3 and in silico 8. We used the networks in silico 3 (a) and in silico 8 (d) to generate data, which was then input to LEM. The most likely regulation of each node (as given by LEM output) was combined to form networks (b,e) with corresponding systems of ODEs. Simulation of the systems of ODEs inferred by LEM was then compared to the original data (c,f). (EPS 1045 kb)

Additional file 21 Figure: Yeast cell-cycle network 1 data and inferred LEM ODE simulations. After running LEM on the yeast cell-cycle data [2], we formed a network (with a corresponding system of ODEs) by selecting the most likely regulation of each node, as evaluated by LEM. The simulation of this system of ODEs was then compared to the original data. (EPS 686 kb)

Additional file 22 Figure: Network diagram for in silico 23. The network consists of a three-node core network with 97 sink nodes added in the same phase. (PDF 8 kb)

Additional file 23 Figure: Network diagram for in silico 24. The network consists of a five-node core network with 95 additional nodes driven by the core. (PDF 12 kb)

Additional file 24 Table: Comparison of methods using AUC-ROC and AUPR scores for the 100-node networks. Scores are reported for the networks in silico 23 and in silico 24 on both the signed and unsigned edge inference challenges. (PDF 31 kb)

Additional file 25 Table: Comparison of methods using MCC scores for the 100-node networks. Scores are reported for the networks in silico 23 and in silico 24 on both the signed and unsigned edge inference challenges. (PDF 29 kb)

Additional file 26 Table: Run times of LEM and the method of Mazur et al. Run times are reported for LEM and the method of Mazur et al. [48] on four in silico networks, each with three nodes. The method of Mazur et al. was run with all default settings, including 10,000 steps of burn-in and 50,000 steps of iteration for the MCMC computations. Although LEM is parallelizable, here we report the amount of time LEM would take to run on a single core. (PDF 34 kb)
