# Supplementary material for: The Local Edge Machine: inference of dynamic models of gene regulation
Source: Genome Biol. 2016 Oct 19;17:214. doi: 10.1186/s13059-016-1076-z (PMC5072315; doi:10.1186/s13059-016-1076-z)
Supplement: Additional file 1 — Supplementary Information. This document contains 13 sections, each of which provides supporting information for some aspect of the study. Additionally, this document contains five internal tables. (PDF 273 kb) [file 13059_2016_1076_MOESM1_ESM.pdf]

SUPPLEMENT TO "THE LOCAL EDGE MACHINE: INFERENCE OF DYNAMIC MODELS OF GENE REGULATION"

KEVIN A. MCGOFF, XIN GUO, ANASTASIA DECKARD, CHRISTINA M. KELLIHER, ADAM R. LEMAN, LAUREN J. FRANCEY, JOHN B. HOGENESCH, STEVEN B. HAASE, AND JOHN L. HARER

1. The model

We consider a gene regulatory network with N nodes $\mathcal{N} = \{X_1, \dots, X_N\}$. For $i = 1, \dots, N$, we let $X_i(t)$ denote the expression level of gene X_i at time t. Our data set, denoted by D, consists of the observed expression levels of the N nodes in $\mathcal{N}$ at T time points, $\{t_j\}_{j=1}^T$. We consider a model space $\mathcal{M}$ as follows. To begin, we suppose that the data are generated according to a system of ordinary differential equations, possibly observed with noise:

$$\frac{d\mathbf{X}}{dt} = F(\mathbf{X}(t)), \tag{1.1}$$

where $\mathbf{X}(t) = (X_1(t), \dots, X_N(t))$. In general, stochastic effects play a significant role in the dynamics of any individual cell, and such considerations lead one to stochastic differential equations. However, we consider data generated by averaging expression levels over many ($\sim 10^8$) individual cells, and we therefore assume that the stochastic effects are insignificant, leading to our adoption of ordinary differential equations.

The behavior of the system in (1.1) is determined by the choice of function $F : \mathbb{R}^N \to \mathbb{R}^N$. For a node X in $\mathcal{N}$, we assume that

$$\frac{dX}{dt} = \alpha f(\mathbf{X}(t)) - \beta X(t) + \gamma, \tag{1.2}$$

where $\alpha > 0$ represents the strength of the regulation, $\beta \geq 0$ represents the rate of degradation of X, and $\gamma \geq 0$ represents the basal rate of production of X. The function $f : \mathbb{R}^N \to \mathbb{R}$ governs the type of regulation that X experiences. Following previous non-linear modeling efforts, we use Hill functions to model the effects of activation and repression in the system. In other words, we say that node A "activates" X, if the function f consists of a term of the form

$$a(A) = \frac{A^n}{A^n + K^n}, \tag{1.3}$$

where $K > 0$ represents the half-level of maximal activation and $n \geq 1$ is called the Hill coefficient. Similarly, we say that A "represses" X, if f consists of a term of the form

$$r(A) = \frac{K^n}{A^n + K^n}. \tag{1.4}$$

The fact that we only consider functions of at most one variable reflects our sparsity assumption that in any given experimental condition, we seek to find the dominant regulatory mechanism for any node.

Given a list of nodes $\mathcal{N}$, we let $\mathcal{L} = \{L_1, \ldots, L_r\}$ be the list of all possible logical regulatory relationships. To each L in $\mathcal{L}$, we associate a parameter space $R_L \subset \mathbb{R}^d_+$

of dimension $d = d(L)$. Hence, the space of possible models for X is given by

$$\mathcal{M}(X) = \bigsqcup_{L \in \mathcal{L}} \{L\} \times R_L,$$

and the full model space for the entire system is

$$\mathcal{M} = \prod_{X \in \mathcal{N}} \mathcal{M}(X).$$

2. Local approximation

Suppose we have a node X, a model $(L, \theta) \in \mathcal{M}(X)$ for X, and data $D = \{X_i(t_j)\}_{i,j}$. Suppose that the logic L involves regulation of X by node A. Then from the data, for each time $t = t_j$ with $j = 1, \ldots, T$, we compute the values

$$F(t) = f_L(A(t)) - \beta X(t) + \gamma,$$

and extend F to the interval $[t_1, t_T]$ by linear interpolation. Then for any t in $[t_1, t_T]$, we analytically integrate to obtain

$$\hat{X}(t) = \int_{t_1}^{t} F(t)\, ds. \tag{2.1}$$

The values $\hat{X}(t)$, properly shifted, represent the predicted values of $X(t)$ made by the model (L, θ) from the data. Notice that this prediction is “local,” in the sense that it refers only to the model for X and not the full (“global”) system of differential equations. From a statistical point of view, this local approximation suffers from a version of the “errors-in-variables” problem, while the global model does not. Nonetheless, it is precisely this local approximation that renders inference of the local model computationally tractable and scalable to moderately large networks.

3. Inference

In this section we describe our inference methodology. Given a node set $\mathcal{N}$, a node X in $\mathcal{N}$, a model $(L, \theta) \in \mathcal{M}(X)$, and data D, we define a loss function

$$\ell(D, L, \theta) = \min_{c \in \mathbb{R}} \frac{1}{T} \sum_{t=1}^{T} (X(t) - \hat{X}(t) - c)^2,$$

where $\hat{X}$ is the (shifted) prediction of $X(t)$ made by the model (L, θ) from data as in (2.1).

Let π be a probability distribution on $\mathcal{M}(X)$, which we view as a prior distribution in a Bayesian framework. The Gibbs posterior principle can be shown to be the optimal (rational) method for updating belief distributions when a fully generative model is unknown.[33] Since we do not attempt to model the noise in the data (which would depend heavily on the technology used, *e.g.* arrays versus sequencing), we are in such a setting. Then by the Gibbs posterior principle,[33,34] we obtain that the posterior distribution on the model $(L, \theta) \in \mathcal{M}(X)$ given the data D is

$$p(L, \theta \mid D) = \frac{\exp\Big(-\ell(D, L, \theta)\Big)\pi(L, \theta)}{\sum_{L' \in \mathcal{L}} \int_{R_{L'}} \exp\Big(-\ell(D, L', \theta')\Big)\pi(L', d\theta')}.$$

Define

$$C(D) = \sum_{L' \in \mathcal{L}} \int_{R_{L'}} \exp\Big(-\ell(D, L', \theta')\Big)\pi(L', d\theta').$$

Then

$$p(L \mid D) = \frac{1}{C(D)} \int_{R_L} \exp\Big(-\ell(D, L, \theta)\Big)\pi(L, d\theta). \tag{3.1}$$

To formulate our prior distribution, we place *a priori* bounds on the real-valued parameters that may appear in any equation. First, we linearly rescale the time so that $t_1 = 0$ and $t_T = 100$. Next, we linearly rescale the expression levels of each node so that they lie in the interval $[0, 100]$ and obtain the maximum value of 100. Our *a priori* bounds are made on this scale. We place bounds on each type of parameter independently; see Supplementary Information Table 1. These bounds were chosen by considering the loose biological interpretation of each parameter and selecting large parameter ranges that include all biologically reasonable speeds of transcription and protein decay. The bounds on α and γ ensure that the maximum slope (transcription rate) is approximately 100. The bounds on β ensure that the minimum slope (decay rate) is -100. The bounds on K ensure that the critical value of the activity of any node is not greater than the maximum of the node. The bounds on n reflect the range of non-linearity that we consider reasonable in these systems.

By choosing these bounds, we have defined a rectangle R in $\mathbb{R}^5_+$. With these bounds in place, we choose the maximum entorpy distribution as our prior as follows:

$$\pi(L, d\theta) = \frac{1}{s}\frac{d\theta}{\mathrm{Vol}(R)},$$

where s is the number of regulatory relationships that have X as the target, $d\theta$ is Lebesgue measure restricted to R, and $\mathrm{Vol}(R)$ is the volume of R.

With these choices, the Gibbs posterior probability of L given D is

$$\begin{aligned} p(L \mid D) &= \frac{1}{sC(D)\,\mathrm{Vol}(R)} \int_R \exp\Big(-\ell(D, L, \theta)\Big)\, d\theta \\ &= \frac{\exp\Big(-\ell(D, L, \theta^*)\Big)}{sC(D)\,\mathrm{Vol}(R)} \int_R \exp\Big(-\ell(D, L, \theta) + \ell(D, L, \theta^*)\Big)\, d\theta, \end{aligned}$$

where θ^* is a minimizer of the function $\theta \mapsto \ell(D, L, \theta)$. The Laplace approximation is a well-known approximation to an integral of the form $\int \exp(f(x))dx$, where f is twice-differentiable.[35] We take f to be the function $\theta \mapsto -\ell(D, L, \theta)$, and note that it is twice-differentiable, since the prediction $\hat{X}(t)$ is twice-differentiable with respect to the parameters in θ. We then use a Laplace approximation to the integral[35] on the right-hand side of the previous display and get

$$\begin{aligned} p(L \mid D) &= \frac{\exp\Big(-\ell(D, L, \theta^*)\Big)}{sC(D)\operatorname{Vol}(R)} \int_R \exp\Big(-\ell(D, L, \theta) + \ell(D, L, \theta^*)\Big) d\theta \\ &\approx \frac{\exp\Big(-\ell(D, L, \theta^*)\Big)}{sC(D)\operatorname{Vol}(R)} \frac{1}{2^{d^*}} \sqrt{\frac{(2\pi)^5}{\det H}}. \end{aligned}$$

Here, H is the Hessian of the map $\theta \mapsto \ell(D, L, \theta)$ at the point θ^*, and d^* is the number of parameters in θ^* whose value is at one of the extremes of the allowed ranges (see Supplementary Information Table 1). Thus, our approximate Gibbs posterior distribution is formed by taking

$$p(L \mid D) \propto \frac{1}{2^{d^*}} (\det H)^{-1/2} \exp\Big(-\ell(D, L, \theta^*)\Big). \tag{3.2}$$

Observe that if the regulation L is robust to changes in parameters near θ^*, then $\det H$ is small, which increases the posterior probability of L. This relationship reflects the widespread belief that evolved, biological networks ought to be robust to changes in parameters.[55] Also, the term 2^{d^*}, which appears in the denominator, decreases the posterior probability of L as the number of extremal parameter values in θ^* increases. The effects of using a Bayesian method (as opposed to simply minimizing the loss function) and estimating the integral over parameter space enter the posterior

through the presence of $\det H$ and d^*, both of which arise directly as a result of our approxmations of the integral.

3.1. **Output of inference.** Given a set $\mathcal{N}$ consisting of N nodes, LEM will generate a set of $2N^2$ logical regulatory relationships (edges): $\{(X, Y, u) : X, Y \in \mathcal{N}, u \in \{a, r\}\}$. For each edge L, LEM computes the right-hand side of (3.2). Then, for each node X, these values are normalized so that the sum over all edges with X as the target is one. Thus, for each node X, LEM returns a probability distribution over the set of possible regulatory controls of X. In order to obtain a probability distribution on the full network, one may take the product of all the node-wise distributions. From such a distribution, there are multiple ways to select a single network. One could select a threshold $0 < \delta < 1$ and declare that any edge L such that $p(L \mid D) \geq \delta$ is in the network.

4. Optimization and computational details

After the local approximation to the model and the inference method outlined in Section 3, it remains to find parameter values θ^* that minimize the loss function $\ell(D, L, \theta^*)$ and compute the Hessian of the loss function at θ^*. Here we must deal with a constrained, non-convex optimization problem, which is computationally difficult. However, due to our sparsity assumption and our local approximation, we need only consider N independent optimization problems on $\mathbb{R}^5_+$. For comparison, the full global problem *in which the edges are already known* would require solving a single optimization problem in $\mathbb{R}^m$, where $m = \Theta(N)$. If the edges were not known in advance, then the global problem involves comparison of $e^{\Theta N^2}$ different network

topologies. However, in our formulation, we have many small optimization problems, which is ideally suited to parallelization.

Our general optimization procedure involves repeated, random selection of parameters θ in R_L, followed by some greedy algorithms from each such selection. Our implementation uses some existing optimization tools in `R` (the function `optim` with algorithm `L-BFGS-B` in `R` package `stats`, and the function `nloptr` in the `R` package `nloptr` with algorithm `NLOPT_LD_SLSQP`).

As LEM must solve a local optimization problem for each possible edge in the network, the amount of computation required by LEM scales quadratically with the number of nodes, *i.e.*, it is $\mathcal{O}(N^2)$.

In practice, LEM is run on a cluster of computers (called the Duke Shared Cluster Resource, or DSCR). For evaluation of the running time, we restricted LEM to run on a small group of computers with CPU clock from 2GHz(Turbo 2.8GHz) to 2.6GHz(Turbo 3.4Hz) and memory from 128GB to 256GB. Note that each machine runs 32 threads of LEM, and by design, LEM is not a memory intensive algorithm. To get a feeling for the running time of a sample computation, see Supplementary Information Table 2, where the run time is the length of time LEM would take to compute the posterior distribution of each node if run on a single, one-thread, CPU core, and Supplementary Information Table 3, where the run time is the length of time LEM would take to compute all posterior distributions (one for each node) if run on a single, one-thread, CPU core.

Given the increasingly wide availability of computer clusters for research in systems biology, we also describe the parallelized time performance of LEM on the DSCR. For example, consider the network "Yeast cell-cycle 5 (replicate 2)". This network

contains 28 nodes, and the total time LEM uses is 24514.531 seconds. However, LEM hires 1568 separate processes to complete these computations, and among these individual processes, the longest time spent is 27.72 seconds. This time is longer than Granger Causality takes, but it is much shorter than the time used by Inferelator, Hill-DBN, Jump3, and TD-ARACNE.

5. Scoring and comparison of algorithms

In order to compare LEM to other algorithms, we use three well-established methods of network comparison, the area under the receiver operating characteristic (AUC-ROC or just AUC) scores,[56] area under the precision-recall curve (AUPR) scores, and Matthew's Correlation Coefficient (MCC).

If an algorithm (such as LEM) produces a ranked list of predictions as output in response to a binary classification problem, then AUC-ROC may be used as assessment of the quality of the predictions. To do so, one plots the true positive rate against the false positive rate after each possible cut-off within the ranked list, which produces the receiver operating characteristic (ROC) curve. The AUC-ROC score is then defined as the area under the ROC curve. A score of 1 indicates perfect classification, whereas a score of 0.5 is the expected score when predictions are made at random (in which case, the ROC curve would be the diagonal). The AUPR is defined similarly using the precision-recall curve in place of the ROC curve; however, in this case, 0.5 is no longer the expected score when predictions are made at random.

Given a "gold standard" network, we interpret the list of edges as the goal for classification: the perfect algorithm would predict all of the "gold standard" edges and not predict any other edges. Since LEM returns a list of probabilities associated

to each possible edge, we use these probabilities to rank-order the LEM predictions, from highest to lowest probability. Since LEM assigns a probability to each signed, directed edge, we compute AUC-ROC and AUPR scores for the signed, directed classification problem (classify all possible signed, directed edges). To assess LEM using the unsigned, directed classification problem, we assign each edge a score equal to the maximum of the two probabilities given to the two signed versions of that edge. Then we rank-order the edges by these scores and again compute AUC-ROC and AUPR scores.

To compare LEM to the Inferelator, we use the recent Inferelator implementation[18] (version 2013.3.RC3). This package includes the option to output ranked lists of predictions, and we therefore compare it to LEM using AUC-ROC and AUPR scores. In this implementation of the Inferelator, the ranked lists of predictions do not include a prediction about the sign of an edge. In order to compare with LEM on the signed classification problem, we assign both the activator and the repressor version of each edge the same score as output by the Inferelator. These scores are all non-negative. In order to incorporate prior information, we simply assign a score of zero to any edge with prior probability zero.

To compare LEM to Granger Causality,[14] we use an implementation in R (package MSBVAR, version 0.9-2). This method requires a choice of a “lag” parameter. We compute the AUC-ROC scores for this method with all possible lags and then report the maximal score. Similarly, we compute the AUPR scores for this methods with all possible lags and report only the maximal score. Note that the maximal AUC and AUPR scores may not correspond to the same lag. For this reason, the scores for Granger Causality should be interpreted as “best possible” and do not necessarily

reflect the results of any single choice of lag. As with the Inferelator, the output of Granger Causality is unsigned, directed edges, and we assign both the activator and the repressor version of each edge the same score as output by the algorithm in the signed classification problem. Also, we enforce that edges with prior probability zero are assigned a p-value of 1.

The algorithm labeled Hill-DBN[22] was evaluated using the MATLAB implementation provided with the paper. All parameters were set to their default or suggested values, including the "maximum number of parents" that any node may have, which was set to 4.

The Jump3 algorithm[25] was executed using the implementation MATLAB implementation published with the paper. All parameters were set to their default or suggested values, including the "system noise" and "observation noise," which were set as in the example provided in the documentation.

To compare LEM to TD-ARACNE and Banjo, we use the a recent TD-ARACNE implementation[19] (R package version 1.22.0) and the most recent Banjo implementation[20,21] (version 2.2.0). Banjo involves a parameter that controls how long it may search through network space. In choosing this parameter, we allowed Banjo to run for twice as long as LEM took on each network. Since these methods are binary classifiers, we use MCC as our basis of comparison. We have also included ROC plots of the LEM results for the networks in Table 1, plotted together with the TD-ARACNE and Banjo results (see Additional File 4). When prior information is included, we enforce that any edge with prior probability zero is not included in the output network.

To perform the binary classification task for LEM (for computation of MCC), we threshold the posterior probability of each edge using a threshold of 0.4 (so an edge e is predicted by LEM if $p(e \mid D) \geq 0.4$). The choice of 0.4 as our threshold was made by testing a range of possible values on the *in silico* datasets, but we readily acknowledge that this choice is somewhat arbitrary and may be altered to fit the needs of the user.

6. Noise and incompleteness testing

Here we present our results regarding the performance of LEM with noise-corrupted data. We use an observational noise model with Gaussian noise at several scales. To be more specific, suppose we have the data $\{X(t_j)\}_{i=1}^{T}$ for node X. We define the signal level σ_{signal} as

$$\sigma^2_{\mathsf{signal}} = \frac{1}{T-1}\sum_{i=1}^{T}(X(t_i) - \bar{X})^2,$$

where $\bar{X} = \frac{1}{T}\sum X(t_i)$ is the average value of X. For a fixed noise scale α, we generate independent Gaussian noise

$$e_i^0 \sim \mathcal{N}(0, (\alpha\sigma_{\mathsf{signal}})^2), \text{ for all } i = 1, \cdots, T.$$

The noise variables are then projected to the 3σ interval

$$e_i = \begin{cases} 3\alpha\sigma_{\mathsf{signal}}, & \text{when } e_i^0 > 3\alpha\sigma_{\mathsf{signal}}, \\ -3\alpha\sigma_{\mathsf{signal}}, & \text{when } e_i^0 < -3\alpha\sigma_{\mathsf{signal}}, \\ e_i^0, & \text{otherwise.} \end{cases}$$

Finally, the testing data we use is given by $\{\max(X(t_i) + e_i, 0.001)\}_{i=1}^T$. This projection is necessary, because a gene expression level can never be negative and LEM is designed accordingly.

In Additional file 19, we show the AUC-ROC scores that LEM obtains under several noise scales and with access to incremental amounts of prior information on several *in silico* networks. In each case, we added noise as above, and also gave LEM access to some fraction of the possible pieces of prior information. The possible prior information was simply an "identity" for each node, which was one of "activator," "repressor," "both/unknown," or "neither." As expected, the performance of LEM degrades under increasing noise and improves as additional prior information is available.

In Additional file 14, we present the AUC-ROC scores that LEM obtains for the yeast cell-cycle networks with increasing amounts of prior information.

7. Non-periodic data

In this section, we describe LEM's performance on four non-periodic datasets. The datasets were generated *in silico*, using an evolutionary algorithm as described in Section 9. However, in these instances, we did not select for periodicity. The AUC-ROC scores for LEM on these networks are given in Supplementary Information Table 4. Note that this performance is comparable to LEM's performance on our oscillating *in silico* datasets with the same numbers of nodes (see Additional file 5).

8. Identifiability

In this section, we present two examples of non-identifiability of network structures. *In silico* networks 3 and 8 are used to generate time-series data. In each case, LEM

returns a "maximum *a posteriori*" network with parameters (obtained by combining the most likely regulation of each node into a system of ODEs). In both cases, the LEM networks are distinct from the generating networks (see Additional file 20). In particular, nodes C and D are under complex regulation in the generating networks, but LEM finds simpler networks. Nonetheless, the solutions of systems of ODEs inferred by LEM matches the data very well (see Additional file 20), highlighting the issue of non-identifiability.

9. Benchmark *in silico* datasets

An evolutionary algorithm was used to create the *in silico* datasets. The algorithm allows "mutations" in parameter values in the differential equations corresponding to a fixed network structure. Selection is based on whether the system of differential equations generates oscillating (periodic) behavior.

A wide variety of such parameterized systems of equations was created by varying the size, logical structure, and complexity of the network used as input by the evolutionary algorithm. To model AND gates such as $(X\&Y) \to Z$, we used equations of the form

$$\frac{dZ}{dt} = \alpha \frac{X^{n_1}}{K_1^{n_1} + X^{n_1}} \cdot \frac{Y^{n_2}}{K_2^{n_2} + Y^{n_2}} - \beta Z + \gamma.$$

Similarly, for an OR gate such as $(X \mid Y) \to Z$, we used equations of the form

$$\frac{dZ}{dt} = \alpha_2 \frac{X^{n_1}}{K_1^{n_1} + X^{n_1}} + \alpha_2 \frac{Y^{n_2}}{K_2^{n_2} + Y^{n_2}} - \beta Z + \gamma.$$

All of our benchmark *in silico* datasets involved about 50 time points, sampled at regular intervals. For the cyclic datasets, we sampled over two cycles, yielding about

25 samples per cycle. This sampling rate is currently on the high end of what is practical in wet lab experiments (see[57] for example).

10. Yeast Cell-cycle datasets

Here we describe the yeast cell-cycle datasets.[2] Wild-type budding yeast cells (strain derivative of BF264-15Dau) were synchronized in the cell cycle, and periodic gene expression profiles were obtained by microarray profiling in two biological replicates (GEO accession number GSE8799). Gene expression data from genes manually curated to be part of a transcription factor (TF) network were used in this study (Additional file 7). The data cover 15 time points over approximately two cell cycles.[2] We removed the stress response (due to synchronization and media shift) and used only the last 13 time points as gene expression data. Eliminating the first two time points gives us the cell-cycle regulated transcription from the experiment.

To generate the heatmap of our cell-cycle genes of interest (Figure 1), a cubic spline interpolation was applied to the gene expression data from replicate 1 to generate expression profiles with 40 data points. Next, we utilized a visualization tool produced by Rescon Ltd. (`www.rescontechnologies.com`) to rank-order the splined gene expression data according to timing of 50% of the peak value (Additional file 7). The Rescon visualization tool also applies a three-color scale by moving-average normalization to indicate the gene expression value at a given data point relative to its moving-average mean. The splined profile of each gene was processed by a moving-average normalization, where the expression value at each point was adjusted to a baseline generated by the average value of up to 16 (±8) of the surrounding data points.

11. Manual curation of yeast cell-cycle networks

For the purpose of testing LEM, we created five different yeast cell-cycle networks, called Yeast cell-cycle networks 1 through 5 (see Additional files 10 through 12). Networks 2 and 3 were derived from previously published cell-cycle networks that are capable of oscillations in a Boolean framework.[2,3] To build complexity, Networks 1 and 4 were obtained by modifying networks 2 and 3 (respectively) to include high confidence targets of transcription factors as sink nodes in the network models (see Additional files 3 and 8). Network 5 is the most expansive model, including all previously identified transcription factors that likely play a role in driving cell-cycle regulated transcription. The 28 nodes and edges in Network 5 are supported by literature and database evidence (Additional files 3 and 8). Evidence and citations for all edges appears in Additional file 3.

12. Circadian datasets and analysis

We analyzed circadian transcript data from 12 mouse tissues provided by the Hogenesch lab.[44] First, as we have previously recommended, we identified periodic genes with periods of 20 to 28 hours using multiple periodicity algorithms, in this case the Lomb-Scargle and JTK_CYCLE periodicity detection algorithms.[58] Observing that JTK_CYCLE captures more of the peaked-type data prevalent in the circadian transcription data, we declared periodic transcripts to be those with a p-value less than 0.1 according to the JTK_CYCLE algorithm. We selected a conservative cutoff for circadian periodicity by aiming for slightly less than 40% of all mouse genes, which was the estimate provided by the Hogenesch group for genes under circadian control. This filter identified circadian periodic transcripts numbering between 1927

and 5498, depending on the tissue (see Supplementary Information Table 5). Next, we identified a set of "core" circadian regulatory network nodes that can be called central circadian regulators with high confidence (see Additional file 15).

To determine regulators of the core, we applied LEM to identify periodic genes that regulate the aforementioned high confidence nodes in multiple organs. LEM generated probabilities for each potential edge from potential regulators to the high confidence core nodes. We summed the LEM output probabilities across all tissues to generate a probability across the entire mouse circadian clock. If an edge was missing in a tissue because the regulator did not meet the periodic cutoff in that tissue, it was scored as a zero. To this sum of probabilities we applied a cutoff of 0.1 to identify regulators. This analysis yielded 333 unique regulators of the high confidence core.

Next, we took this list of candidates (and the high-confidence core set) and ran LEM to determine whether the high-confidence core regulated the candidate circadian regulators. Rather than use all of the tissues, we restricted this analysis to the liver circadian data that was considered periodic to reduce the complexity of the output results. The 333 unique regulators together with the core genes yielded a set of 354 genes, of which 205 were either known core genes or considered periodic in the liver circadian data set. These 205 gene expression profiles from liver were then run through LEM. To rank these candidate circadian genes, we created a measure of the likelihood of a "core-to-X-to-core" relationship: the maximum LEM probability that the candidate was a regulator of any core element multiplied by the maximum LEM probability that the candidate was regulated by any high-condence core element in the liver. These results are listed in Additional file 16.

13. Experimental validation of circadian analysis

13.1. **Cell culture and reverse siRNA transfection.** Mouse NIH3T3 fibroblast cells were maintained in DMEM (Gibco) supplemented with 10% FBS (HyClone) and 1x penicillin-streptomycin-glutamine (PSG; Invitrogen) and incubated at 37°C, 5% CO2. For transfection experiments, pooled siRNA mixes (n=4/gene; Qiagen) were diluted to a final concentration of 24 nM in transfection medium (Opti-MEM I Reduced Serum Medium (Gibco) supplemented with 2% Lipofectamine RNAiMAX (Life Technologies)). siRNA pools are incubated with the transfection medium for 20 minutes at room temperature to form liposome complexes. Cells suspensions were prepared at a density of 67,000 cells/ml in DMEM supplemented with 20% FBS (HyClone), 2x-glutamine (Invitrogen) without antibiotics. Primed siRNA-liposome complexes were added to cells drop-wise. Cells were seeded with a multichannel into 96-well plates at a final concentration of 10,000 cells/well with a final pooled siRNA concentration of 8nM. Transfected cells were incubated for 24 hours at 37°C, 5% CO2 prior to use in kinetic bioluminescence assays.

13.2. **Bioluminescence recording and data analysis.** siRNA/transfection medium was aspirated 24 h post–transfection and replaced with bioluminescence recording medium (phenol red–free DMEM (Sigma D-2902), sodium bicarbonate (Invitrogen), D-(+)-glucose (Sigma), 10mM HEPES (Invitrogen), 1% pen/strep/L-glutamine (Invitrogen), 0.1mM luciferin (Promega)) supplemented with 0.1 uM dexamethasone (Sigma-Aldrich) to synchronize the cells and provide substrate for the Per2:Luc reporter prior to luminescence recordings. Bioluminescence was recorded at a 1h resolution for 6 days at 32°C using a Synergy 2 (BioTek) microplate reader. All

transfections were performed in triplicate for bioluminescence recordings. Period length quantification was done using the Waveclock package (Price et al., 2008) implemented in R. Data were plotted in Excel using mean bioluminescence over time.

13.3. **Reverse transcription and RT-qPCR analysis for siRNA efficiency validation.** Replicate 96 well plates were seeded in parallel to the bioluminescence assay plates in order to validate siRNA knockdown efficiency by real-time quantitative PCR (RT-qPCR) using TaqMan chemistry (Applied Biosystems). Cells were harvested 48-72 h post transfection in Qiazol (Qiagen) and RNA was extracted following the Directzol-96 RNA manufacturer's protocol (Zymo Research). cDNA was prepared from 500 ng RNA following the qScript cDNA Supermix (Quanta BioSciences) protocol. cDNA was diluted 1:5 for all qPCR reactions and amplified using the PerfeCTa FastMix II, Low ROX (Quanta) reagent. RT-qPCR cycling parameters were 95°C for 30 s followed by 45 cycles of 95°C for 5 s and 60°C for 30 s on the Viia7 instrument (Applied Biosystems). All qPCR reactions were performed in triplicate and normalized to Gapdh as an endogenous control. Relative expression was determined using the delta delta CT method normalizing all samples to the Allstars negative siRNA control expression levels.

References

[55] Barkal, N., Leibler, S.: Robustness in simple biochemical networks. Nature **387**(6636), 913–917 (1997)

[56] Egan, J.P.: Signal detection theory and {ROC} analysis (1975)

[57] Bar-Joseph, Z.: Analyzing time series gene expression data. Bioinformatics **20**(16), 2493–2503 (2004)

[58] Deckard, A., Anafi, R.C., Hogenesch, J.B., Haase, S.B., Harer, J.: Design and analysis of large-scale biological rhythm studies: a comparison of algorithms for detecting periodic signals in biological data. Bioinformatics, 541 (2013)

Parameter	Lower bound	Upper Bound	Loose interpretation
α	0	100	maximal transcription rate
β	0	1	degredation rate
γ	0	0.3	basal expression rate
K	0	100	threshold of regulator
n	1	10	Hill non-linearity

Supplementary Information Table 1: **Parameter bounds used by LEM.** As described in Supplementary Information Section 3, LEM rescales both time and the expression levels of each gene individually so that the time course runs from 0 to 100 time units and the maximum expression value attained is 100. After this rescaling, the above bounds are used on the parameters appearing in Equations (1.2) - (1.4) in Supplementary Information Section 1.

Node	Run Time (sec.)
A	236.724
B	239.003
C	252.353
D	259.724
E	219.866
TOTAL	1207.67

Node	Run Time (sec.)
A	451.783
B	459.081
C	426.512
D	464.851
E	424.67
F	414.671
G	432.243
H	488.815
I	451.673
J	439.277
TOTAL	4453.576

Supplementary Information Table 2: **Running times used to compute the posterior distribution of each node in networks *In silico* 3 and *In silico* 18, respectively.** The run times are given in seconds on a single, one-thread, CPU core.

Network	# Nodes	Time (seconds)
In silico 1	3	402.25
In silico 2	3	505.866
In silico 6	3	456.089
In silico 7	3	333.581
In silico 3	5	1207.67
In silico 8	5	1062.454
In silico 9	5	1125.322
In silico 10	5	1027.981
In silico 4	10	3776.992
In silico 11	10	4283.354
In silico 12	10	3824.53
In silico 13	10	4467.378
In silico 14	10	3979.865
In silico 15	10	4046.833
In silico 16	10	3999.285
In silico 17	10	4392.954
In silico 18	10	4453.576
In silico 19	10	4378.909
In silico 5	20	17587.971
In silico 20	20	16947.546
In silico 21	20	16843.079
In silico 22	20	16849.042
Yeast cell-cycle 1 (replicate 1)	17	9226.179
Yeast cell-cycle 1 (replicate 2)	17	8628.105
Yeast cell-cycle 2 (replicate 1)	8	2024.189
Yeast cell-cycle 2 (replicate 2)	8	1936.728
Yeast cell-cycle 3 (replicate 1)	10	2949.304
Yeast cell-cycle 3 (replicate 2)	10	3207.803
Yeast cell-cycle 4 (replicate 1)	19	10783.98
Yeast cell-cycle 4 (replicate 2)	19	11417.906
Yeast cell-cycle 5 (replicate 1)	28	24531.552
Yeast cell-cycle 5 (replicate 2)	28	24514.531

Supplementary Information Table 3: **Running times required to compute all posterior distributions on all networks.** Running times are given in seconds on a single, one-thread, CPU core.

Network	# nodes	LEM AUC-ROC
Non-periodic 1	10	1.0000
Non-periodic 2	10	0.8363
Non-periodic 3	20	0.8170
Non-periodic 4	20	0.8362

Supplementary Information Table 4: **Performance of LEM on non-periodic datasets.** Four non-periodic datasets were generated *in silico*, as described in Supplementary Information Section 7. Here we present the number of nodes in each network, along with the AUC-ROC score earned by LEM on each network.

Tissue	# genes with JTK p-value less than 0.1
adrenal gland	2852
aorta	2306
brown fat	3897
brainstem	1927
cerebellum	2093
heart	2888
hypothalamus	2616
kidney	5156
liver	5396
lung	5498
skeletal muscle	2435
white fat	2125

Supplementary Information Table 5: **Number of gene expression profiles called periodic in each tissue.** The gene expression profiles in 12 different mouse tissues were generated using microarrays.[44] The microarray contained $21,406$ probesets. The expression profiles for each tissue were searched for periods in the range from 20 to 28 hours using JTK-CYCLE. A gene was called periodic in a particular tissue if JTK-CYCLE returned a p-value of less than 0.1 for the gene's expression profile in that tissue.
