# Supplementary figures and images for: The Local Edge Machine: inference of dynamic models of gene regulation
Source: Genome Biol. 2016 Oct 19;17:214. doi: 10.1186/s13059-016-1076-z (PMC5072315; doi:10.1186/s13059-016-1076-z)

**AUC of LEM: 1.0000**

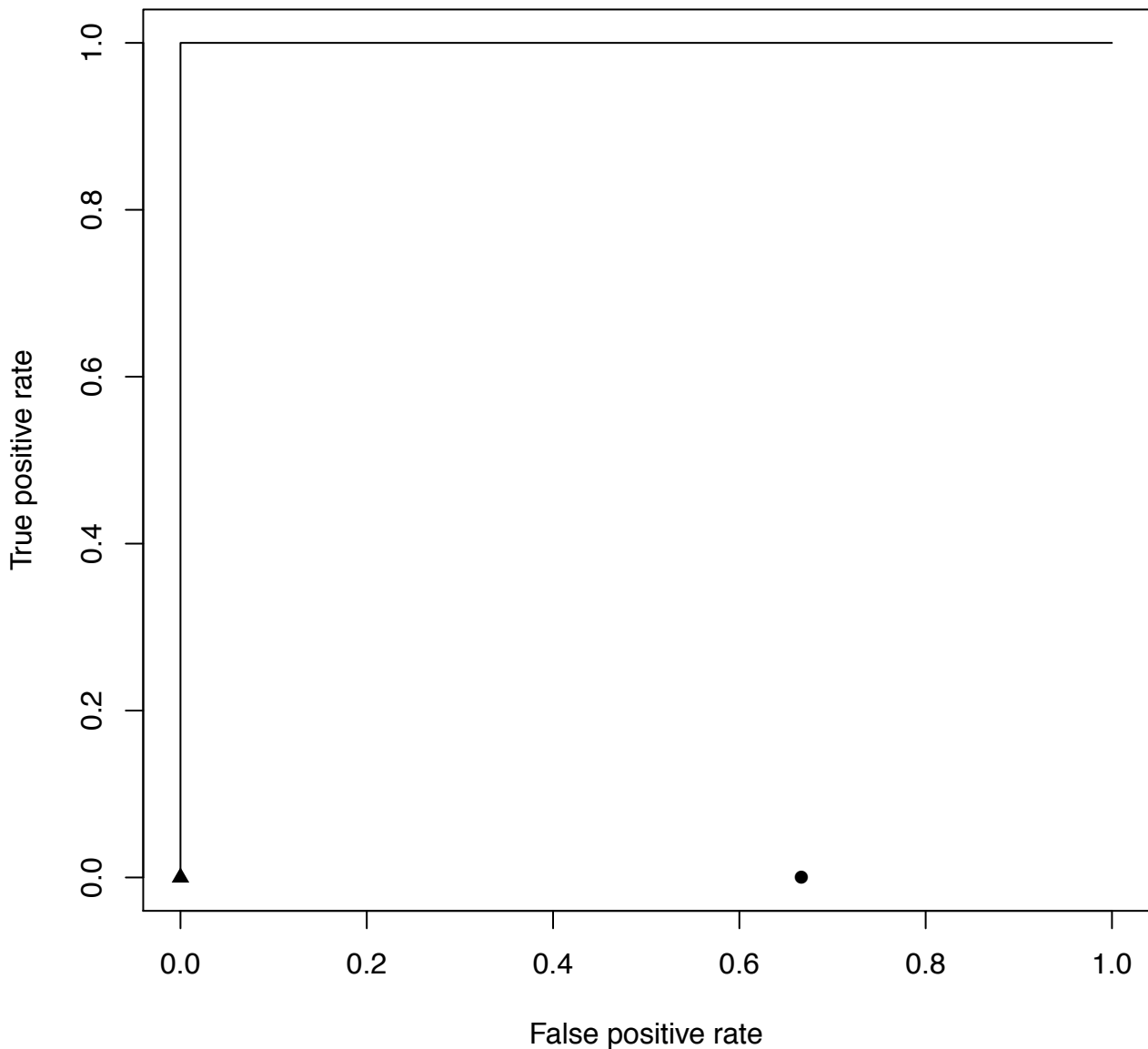

**AUC of LEM: 1.0000**

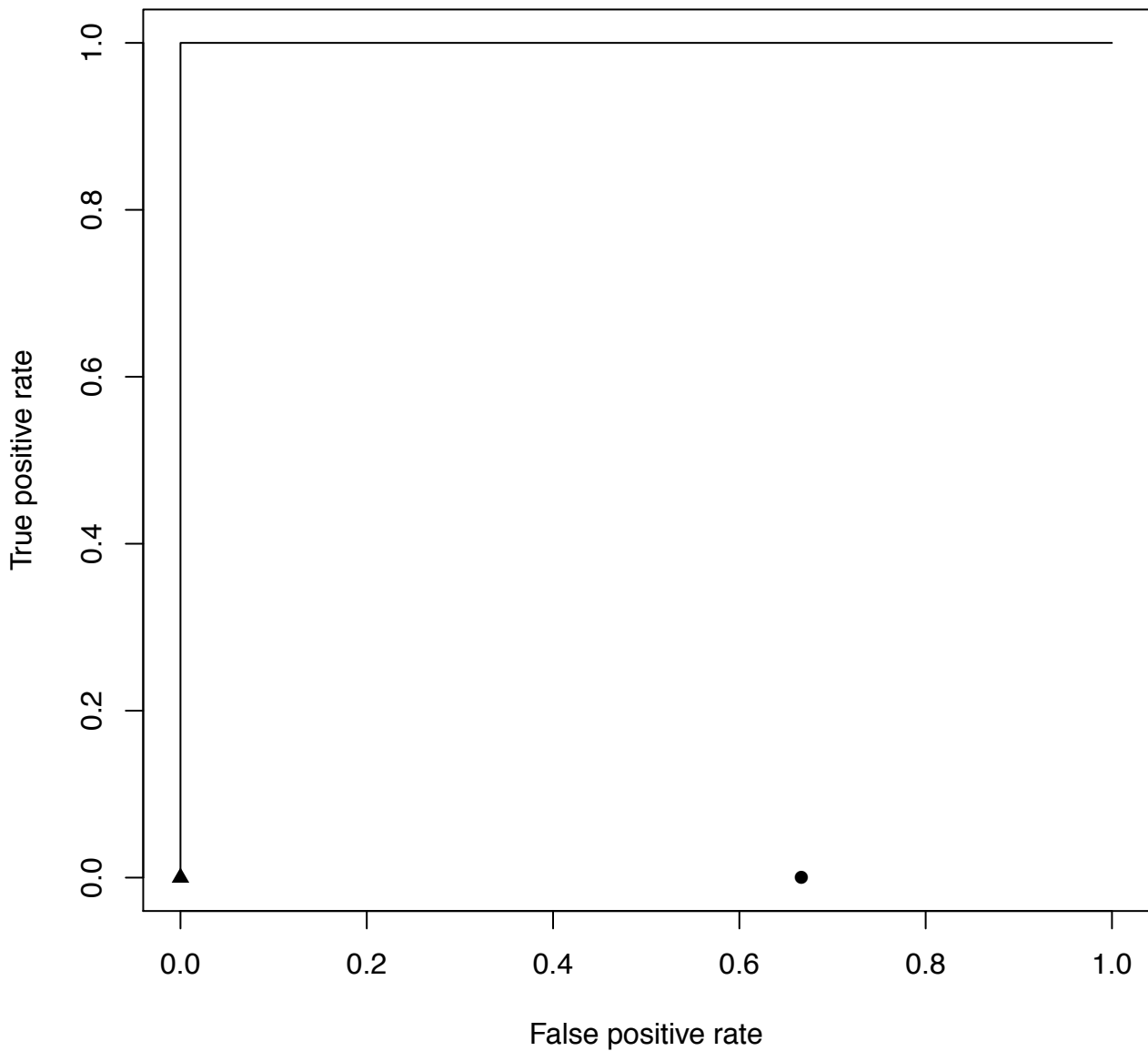

**AUC of LEM: 0.9900**

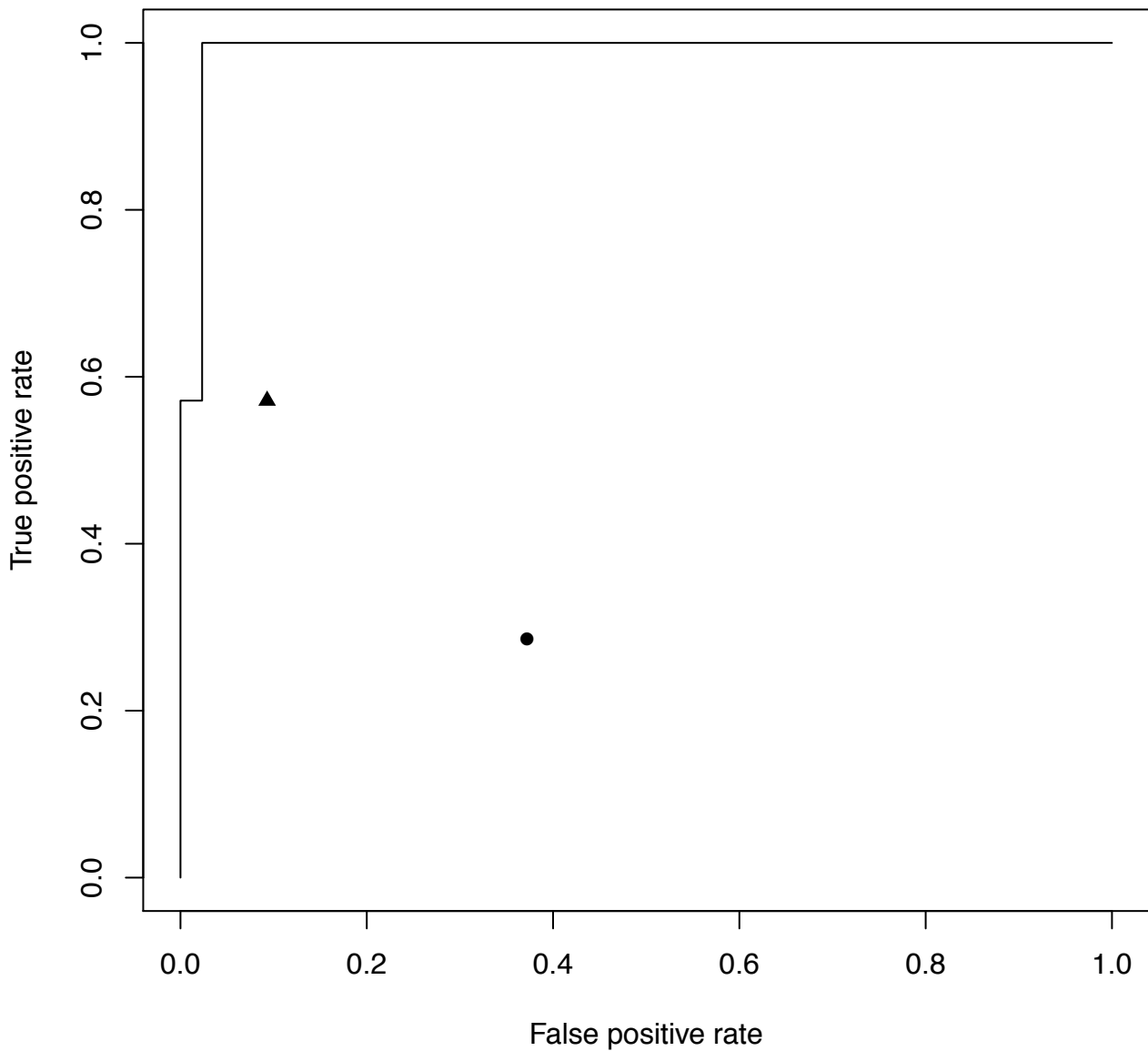

**AUC of LEM: 0.8884**

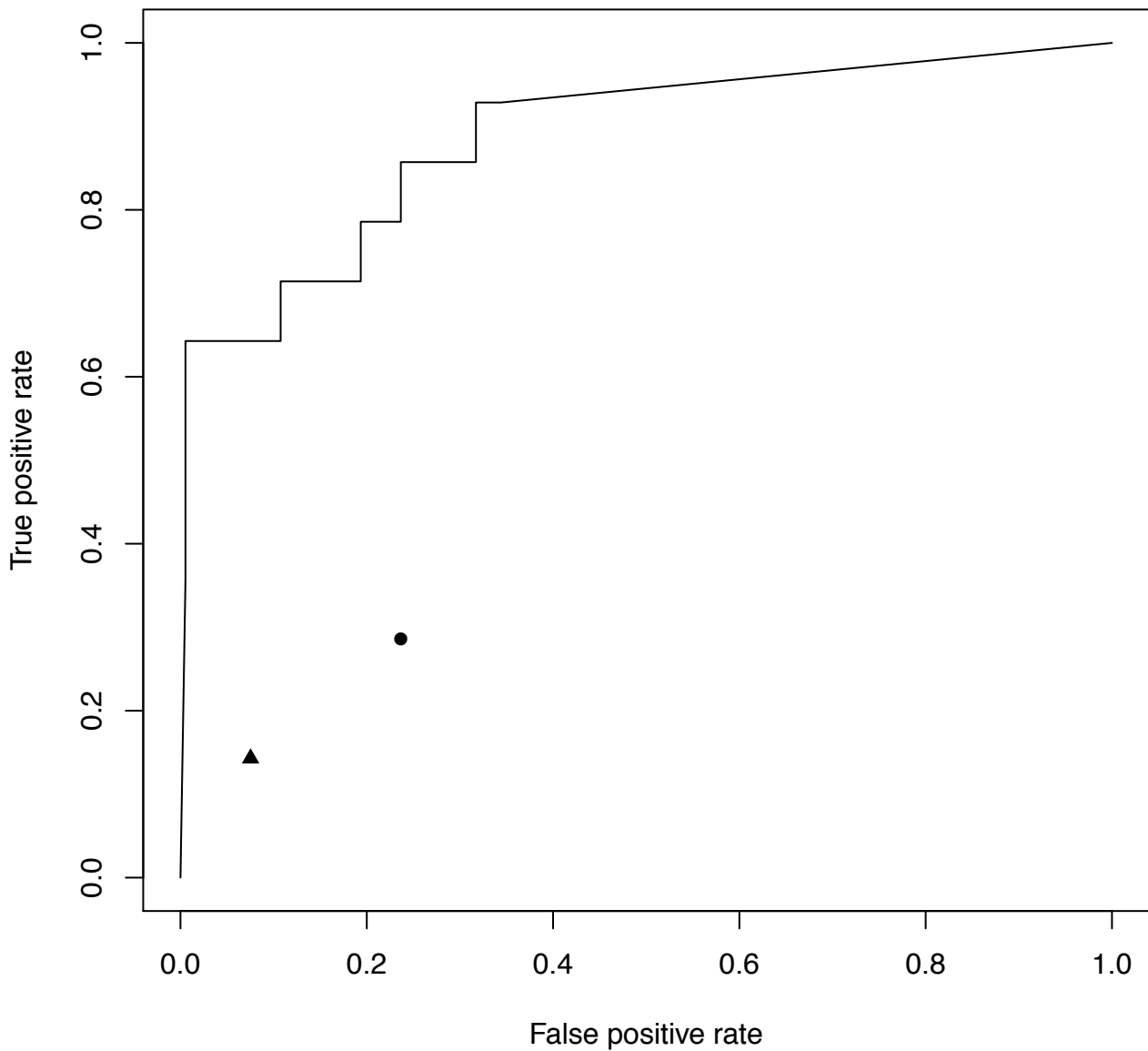

**AUC of LEM: 0.8781**

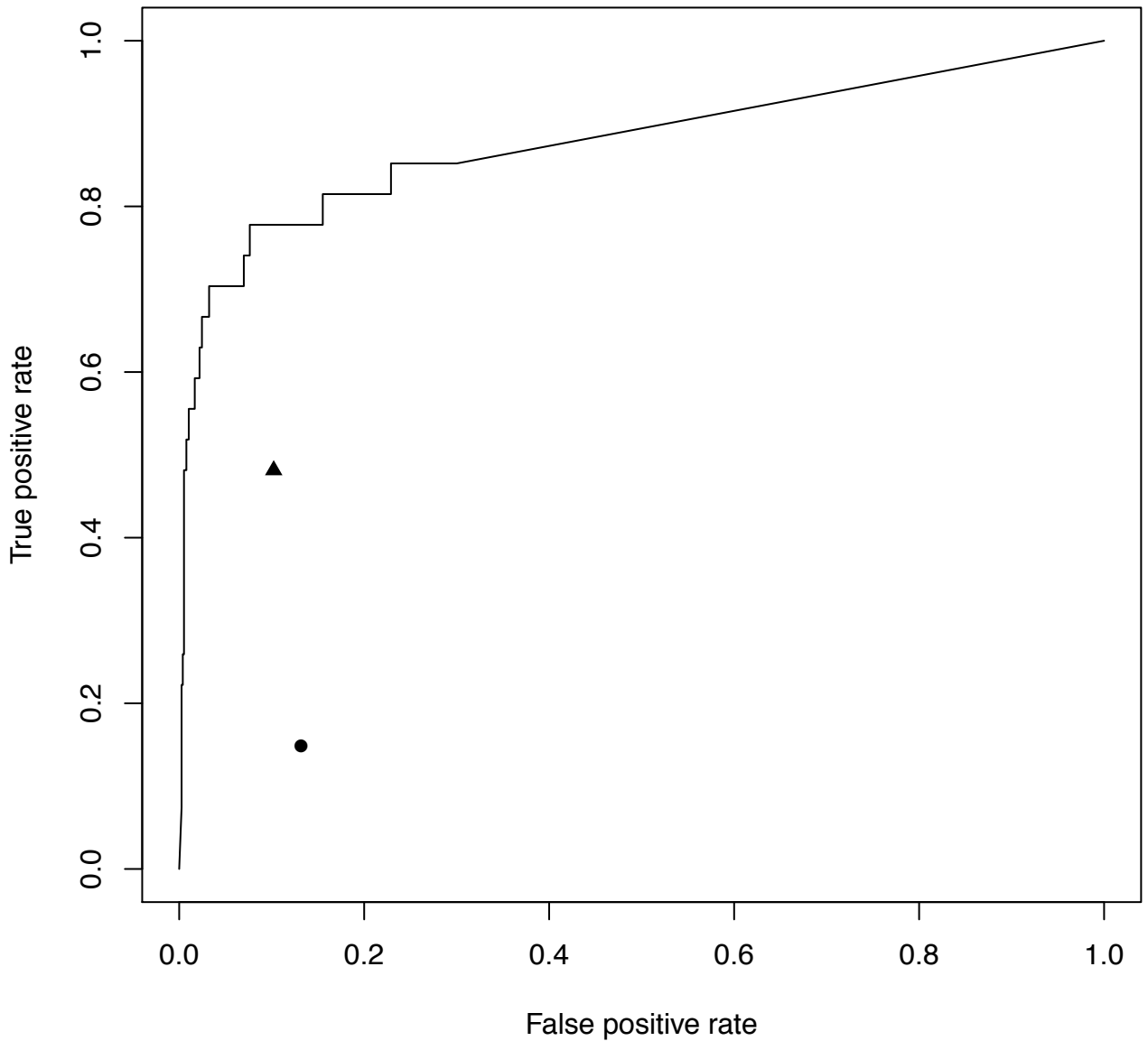

**AUC of LEM: 0.8693**

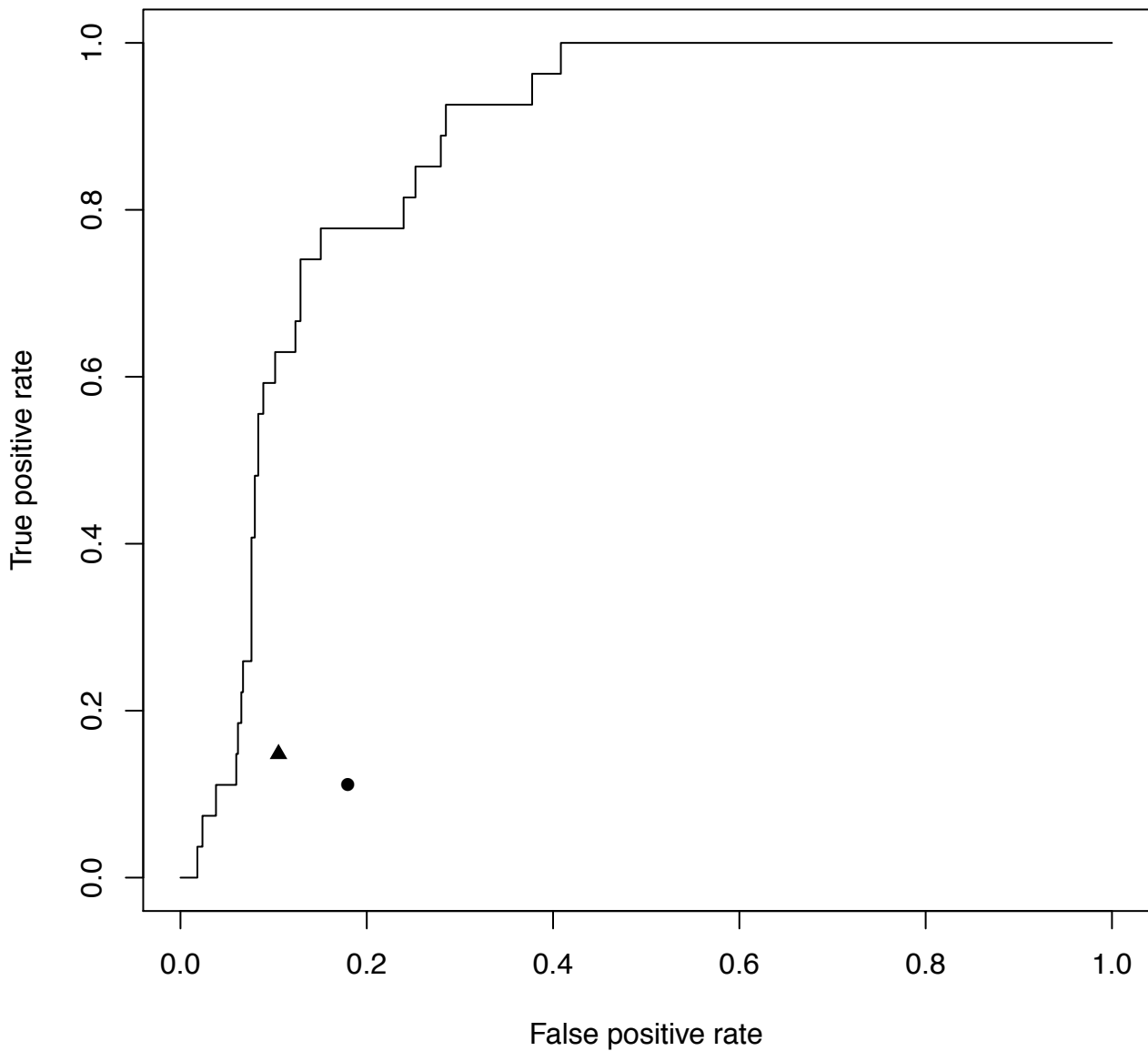

Supplement: Additional file 4 — Figure: ROC plots of LEM together with the results of TD-ARACNE and Banjo. For each of the six networks in Table 1 (in silico 1–5 and yeast cell-cycle network 1), we plotted the ROC curve generated by LEM for the signed directed-edge classification problem. Additionally, we plotted the corresponding results for the binary classifiers TD-ARACNE (marked with a triangle) and Banjo (marked with a square). See Additional file 1: Section 5 for details of these comparisons. (PDF 106 kb) [file 13059_2016_1076_MOESM4_ESM.pdf]

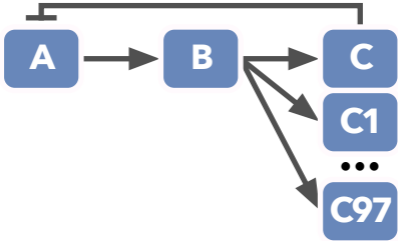

Supplement: Additional file 22 — Figure: Network diagram for in silico 23. The network consists of a three-node core network with 97 sink nodes added in the same phase. (PDF 8 kb) [file 13059_2016_1076_MOESM22_ESM.pdf]

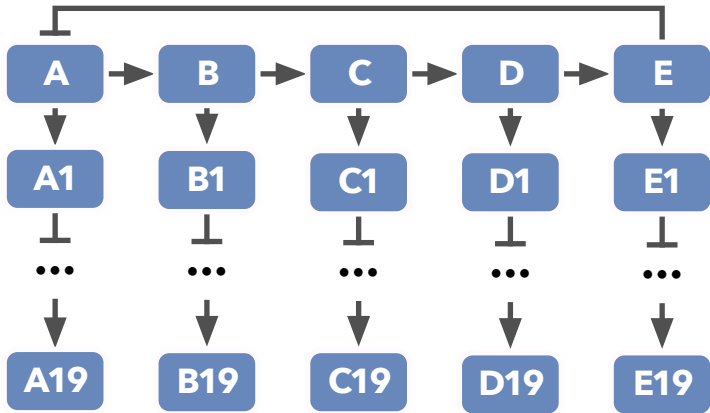

Supplement: Additional file 23 — Figure: Network diagram for in silico 24. The network consists of a five-node core network with 95 additional nodes driven by the core. (PDF 12 kb) [file 13059_2016_1076_MOESM23_ESM.pdf]
