# Supplementary material for: The Local Edge Machine: inference of dynamic models of gene regulation
Source: Genome Biol. 2016 Oct 19;17:214. doi: 10.1186/s13059-016-1076-z (PMC5072315; doi:10.1186/s13059-016-1076-z)
Supplement: Additional file 5 — Table: Comparison of LEM to existing algorithms on both in silico benchmark datasets and in vivo datasets. As described in Additional file 1: Section 5, LEM was compared to Inferelator, Granger Causality, Hill-DBN, and Jump3 using both AUC-ROC scores and AUPR scores and to TD-ARACNE and Banjo using MCC. See Additional file 1: Section 9 for a description of the in silico networks and Additional file 1: Section 11 for a description of the yeast cell-cycle networks. No prior information was used in these comparisons. Replicates 1 and 2 indicate the biological data input to LEM for predictions on yeast cell-cycle networks 1–5. (PDF 44 kb) [file 13059_2016_1076_MOESM5_ESM.pdf]

Network (dataset)	# Nodes	LEM (AUC)	Inferelator (AUC)	Granger Causality (AUC)	Hill-DBN (AUC)	Jump3 (AUC)
In silico 1	3	1.0000	0.9000	0.7000	0.5000	0.9000
In silico 2	3	1.0000	0.5667	0.8111	0.3667	0.7222
In silico 6	3	1.0000	0.8111	0.8556	0.5000	0.7667
In silico 7	3	1.0000	0.8556	0.7444	0.2778	0.8556
In silico 3	5	0.9900	0.7857	0.7791	0.4003	0.6794
In silico 8	5	0.9867	0.6395	0.8787	0.4003	0.8588
In silico 9	5	1.0000	0.6689	0.6600	0.4022	0.6067
In silico 10	5	1.0000	0.9444	0.7844	0.7222	0.6867
In silico 4	10	0.8884	0.5541	0.5949	0.5131	0.7727
In silico 11	10	0.9100	0.6977	0.6771	0.6829	0.7954
In silico 12	10	0.9064	0.6392	0.7491	0.6007	0.7350
In silico 13	10	0.8837	0.6615	0.6737	0.5433	0.7002
In silico 14	10	0.9181	0.6654	0.6569	0.5068	0.8207
In silico 15	10	0.9237	0.7276	0.7255	0.6515	0.7745
In silico 16	10	1.0000	0.6553	0.7568	0.6942	0.8400
In silico 17	10	1.0000	0.8579	0.7368	0.7611	0.9568
In silico 18	10	0.9184	0.7105	0.6723	0.6479	0.7800
In silico 19	10	0.8825	0.6628	0.6402	0.5369	0.7680
In silico 5	20	0.8781	0.6789	0.7441	0.6770	0.7540
In silico 20	20	0.9037	0.7718	0.8235	0.6758	0.7839
In silico 21	20	0.8233	0.6388	0.6429	0.5129	0.6584
In silico 22	20	0.8157	0.6927	0.6933	0.6376	0.7174
Yeast cell-cycle 1 (replicate 1)	17	0.8693	0.6705	0.6893	0.6253	0.6481
Yeast cell-cycle 1 (replicate 2)	17	0.8465	0.6592	0.6766	0.5239	0.5483
Yeast cell-cycle 2 (replicate 1)	8	0.8460	0.6551	0.6032	0.5056	0.6099
Yeast cell-cycle 2 (replicate 2)	8	0.8404	0.6680	0.6300	0.5446	0.5424
Yeast cell-cycle 3 (replicate 1)	10	0.7092	0.6064	0.6224	0.5077	0.6317
Yeast cell-cycle 3 (replicate 2)	10	0.6957	0.6365	0.6022	0.6407	0.5104
Yeast cell-cycle 4 (replicate 1)	19	0.7409	0.5790	0.6333	0.5588	0.6284
Yeast cell-cycle 4 (replicate 2)	19	0.7208	0.6269	0.6319	0.5476	0.5266
Yeast cell-cycle 5 (replicate 1)	28	0.5138	0.5056	0.5407	0.4432	0.5231
Yeast cell-cycle 5 (replicate 2)	28	0.4804	0.5410	0.5836	0.4720	0.5243

Network (dataset)	# Nodes	LEM (AUPR)	Inferelator (AUPR)	Granger Causality (AUPR)	Hill-DBN (AUPR)	Jump3 (AUPR)
In silico 1	3	0.5000	0.4167	0.2139	0.1500	0.4167
In silico 2	3	0.8333	0.1986	0.3417	0.1236	0.2444
In silico 6	3	0.5000	0.3417	0.3681	0.1500	0.2722
In silico 7	3	0.5000	0.3681	0.2306	0.1097	0.3681
In silico 3	5	0.8611	0.3924	0.2841	0.1161	0.2092
In silico 8	5	0.7184	0.2030	0.4164	0.1151	0.3782
In silico 9	5	0.8000	0.2381	0.1379	0.0774	0.1210
In silico 10	5	0.7000	0.4500	0.1833	0.1522	0.1382
In silico 4	10	0.4729	0.0866	0.0933	0.0782	0.1899
In silico 11	10	0.5187	0.2036	0.1481	0.1386	0.2662
In silico 12	10	0.6291	0.1687	0.2501	0.1131	0.2599
In silico 13	10	0.4651	0.1917	0.1468	0.0984	0.1562
In silico 14	10	0.5295	0.2110	0.1327	0.0881	0.2987
In silico 15	10	0.5239	0.2141	0.1663	0.1191	0.2378
In silico 16	10	0.7500	0.1370	0.1392	0.1018	0.1686
In silico 17	10	0.7500	0.2116	0.1193	0.1211	0.4306
In silico 18	10	0.5727	0.2355	0.1820	0.1357	0.2681
In silico 19	10	0.3506	0.1373	0.1180	0.1227	0.2017
In silico 5	20	0.4577	0.0768	0.0719	0.0742	0.0851
In silico 20	20	0.6261	0.1800	0.1368	0.1014	0.1306
In silico 21	20	0.4665	0.1186	0.0817	0.0618	0.0658
In silico 22	20	0.3022	0.0922	0.0733	0.0951	0.0961
Yeast cell-cycle 1 (replicate 1)	17	0.1630	0.0977	0.0877	0.0673	0.0857
Yeast cell-cycle 1 (replicate 2)	17	0.1352	0.0758	0.0737	0.0489	0.0646
Yeast cell-cycle 2 (replicate 1)	8	0.3219	0.1971	0.1764	0.1655	0.2488
Yeast cell-cycle 2 (replicate 2)	8	0.3314	0.2268	0.2106	0.1812	0.1343
Yeast cell-cycle 3 (replicate 1)	10	0.2046	0.1611	0.1782	0.1345	0.2077
Yeast cell-cycle 3 (replicate 2)	10	0.2001	0.2150	0.2060	0.2024	0.1434
Yeast cell-cycle 4 (replicate 1)	19	0.1036	0.0680	0.0879	0.0608	0.0865
Yeast cell-cycle 4 (replicate 2)	19	0.0856	0.0714	0.0732	0.0548	0.0621
Yeast cell-cycle 5 (replicate 1)	28	0.1084	0.1103	0.1091	0.0905	0.1028
Yeast cell-cycle 5 (replicate 2)	28	0.0913	0.1135	0.1201	0.0953	0.1085

Network (dataset)	# Nodes	LEM (MCC)	TD-ARACNE (MCC)	Banjo DBN (MCC)		
In silico 1	3	1.0000	0.0000	-0.5000		
In silico 2	3	1.0000	0.0000	-0.5000		
In silico 6	3	1.0000	0.0000	0.3162		
In silico 7	3	1.0000	0.0000	0.3162		
In silico 3	5	0.7379	0.4528	-0.0624		
In silico 8	5	0.8262	0.2956	0.0235		
In silico 9	5	1.0000	0.4001	0.1361		
In silico 10	5	1.0000	0.3932	-0.1612		
In silico 4	10	0.7463	0.0636	0.0294		
In silico 11	10	0.6342	0.1732	-0.0034		
In silico 12	10	0.7295	0.1382	-0.0271		
In silico 13	10	0.5692	0.0636	0.0439		
In silico 14	10	0.6934	0.2089	0.0624		
In silico 15	10	0.6934	0.2293	0.0214		
In silico 16	10	1.0000	0.3041	0.1934		
In silico 17	10	1.0000	0.1860	0.0443		
In silico 18	10	0.5882	0.0647	0.0890		
In silico 19	10	0.3866	0.0392	-0.0102		
In silico 5	20	0.5908	0.2147	0.0086		
In silico 20	20	0.6982	0.2207	0.0642		
In silico 21	20	0.5285	0.1225	0.0302		
In silico 22	20	0.4657	0.0524	-0.0021		
Yeast cell-cycle 1 (replicate 1)	17	0.0478	0.0292	-0.0380		
Yeast cell-cycle 1 (replicate 2)	17	-0.0385	-0.0045	-0.0145		
Yeast cell-cycle 2 (replicate 1)	8	0.1732	0.0000	-0.0862		
Yeast cell-cycle 2 (replicate 2)	8	0.0976	0.1091	-0.1464		
Yeast cell-cycle 3 (replicate 1)	10	0.2208	0.0388	-0.0032		
Yeast cell-cycle 3 (replicate 2)	10	0.1459	0.1175	-0.0751		
Yeast cell-cycle 4 (replicate 1)	19	0.0422	0.0752	-0.0230		
Yeast cell-cycle 4 (replicate 2)	19	-0.0355	0.0745	-0.0033		
Yeast cell-cycle 5 (replicate 1)	28	0.0018	0.0307	-0.0074		
Yeast cell-cycle 5 (replicate 2)	28	-0.0100	0.0234	-0.0362		
