# Supplementary material for: The Local Edge Machine: inference of dynamic models of gene regulation
Source: Genome Biol. 2016 Oct 19;17:214. doi: 10.1186/s13059-016-1076-z (PMC5072315; doi:10.1186/s13059-016-1076-z)
Supplement: Additional file 6 — Table: Comparison of LEM to existing algorithms on the unsigned directed-edge classification problem. LEM was also compared to existing algorithms on the unsigned directed-edge classification problem, in which the sign of the edge (activator or repressor) is removed from consideration. (PDF 43 kb) [file 13059_2016_1076_MOESM6_ESM.pdf]

Network (dataset)	# Nodes	LEM (AUC)	Inferelator (AUC)	Granger Causality (AUC)	Hill-DBN (AUC)	Jump3 (AUC)
In silico 1	3	1.0000	1.0000	0.7500	0.5000	1.0000
In silico 2	3	1.0000	0.5833	0.8889	0.3333	0.7778
In silico 6	3	1.0000	0.8889	0.9444	0.5000	0.8333
In silico 7	3	1.0000	0.9444	0.8056	0.2222	0.9444
In silico 3	5	0.9762	0.8413	0.8333	0.3810	0.7143
In silico 8	5	0.9683	0.6667	0.9524	0.3810	0.9286
In silico 9	5	1.0000	0.6900	0.6800	0.3900	0.6200
In silico 10	5	1.0000	1.0000	0.8200	0.7500	0.7100
In silico 4	10	0.8559	0.5586	0.6026	0.5141	0.7949
In silico 11	10	0.8583	0.7179	0.6953	0.7016	0.8257
In silico 12	10	0.8679	0.6545	0.7764	0.6118	0.7608
In silico 13	10	0.8103	0.6792	0.6928	0.5481	0.7222
In silico 14	10	0.8661	0.6812	0.6719	0.5074	0.8512
In silico 15	10	0.8743	0.7493	0.7470	0.6659	0.8006
In silico 16	10	1.0000	0.6639	0.7711	0.7050	0.8589
In silico 17	10	1.0000	0.8778	0.7500	0.7756	0.9822
In silico 18	10	0.8753	0.7321	0.6899	0.6630	0.8086
In silico 19	10	0.8339	0.6761	0.6516	0.5399	0.7899
In silico 5	20	0.8267	0.6854	0.7530	0.6834	0.7632
In silico 20	20	0.8729	0.7817	0.8352	0.6822	0.7942
In silico 21	20	0.7598	0.6450	0.6493	0.5135	0.6655
In silico 22	20	0.7607	0.7008	0.7014	0.6434	0.7265
Yeast cell-cycle 1 (replicate 1)	17	0.7409	0.6793	0.6990	0.6318	0.6557
Yeast cell-cycle 1 (replicate 2)	17	0.7000	0.6674	0.6858	0.5251	0.5507
Yeast cell-cycle 2 (replicate 1)	8	0.6849	0.6810	0.6204	0.5065	0.6283
Yeast cell-cycle 2 (replicate 2)	8	0.6823	0.6960	0.6517	0.5521	0.5495
Yeast cell-cycle 3 (replicate 1)	10	0.5720	0.6206	0.6386	0.5087	0.6492
Yeast cell-cycle 3 (replicate 2)	10	0.5515	0.6546	0.6157	0.6594	0.5118
Yeast cell-cycle 4 (replicate 1)	19	0.6035	0.5832	0.6402	0.5619	0.6351
Yeast cell-cycle 4 (replicate 2)	19	0.5791	0.6335	0.6388	0.5501	0.5280
Yeast cell-cycle 5 (replicate 1)	28	0.4403	0.5019	0.5353	0.4345	0.5324
Yeast cell-cycle 5 (replicate 2)	28	0.4678	0.5517	0.5830	0.4621	0.5295

Network (dataset)	# Nodes	LEM (AUPR)	Inferelator (AUPR)	Granger Causality (AUPR)	Hill-DBN (AUPR)	Jump3 (AUPR)
In silico 1	3	0.5000	0.8333	0.4278	0.3000	0.8333
In silico 2	3	0.8333	0.3972	0.6833	0.2472	0.4889
In silico 6	3	0.5000	0.6833	0.7361	0.3000	0.5444
In silico 7	3	0.5000	0.7361	0.4611	0.2194	0.7361
In silico 3	5	0.8611	0.7849	0.5681	0.2322	0.4184
In silico 8	5	0.7184	0.4061	0.8327	0.2302	0.7564
In silico 9	5	0.8000	0.4762	0.2758	0.1549	0.2419
In silico 10	5	0.7000	0.9000	0.3666	0.3044	0.2764
In silico 4	10	0.4977	0.1732	0.1865	0.1564	0.3797
In silico 11	10	0.5302	0.4072	0.2961	0.2772	0.5324
In silico 12	10	0.6480	0.3374	0.5003	0.2262	0.5197
In silico 13	10	0.4777	0.3834	0.2937	0.1967	0.3123
In silico 14	10	0.5334	0.4219	0.2654	0.1762	0.5974
In silico 15	10	0.5273	0.4282	0.3327	0.2382	0.4756
In silico 16	10	0.7500	0.2740	0.2783	0.2037	0.3372
In silico 17	10	0.7500	0.4231	0.2386	0.2421	0.8613
In silico 18	10	0.5819	0.4711	0.3640	0.2713	0.5361
In silico 19	10	0.3895	0.2745	0.2360	0.2454	0.4033
In silico 5	20	0.4602	0.1536	0.1438	0.1485	0.1701
In silico 20	20	0.6312	0.3599	0.2735	0.2029	0.2613
In silico 21	20	0.4763	0.2372	0.1634	0.1235	0.1317
In silico 22	20	0.3155	0.1845	0.1466	0.1902	0.1922
Yeast cell-cycle 1 (replicate 1)	17	0.1659	0.1954	0.1755	0.1345	0.1713
Yeast cell-cycle 1 (replicate 2)	17	0.1398	0.1517	0.1474	0.0978	0.1291
Yeast cell-cycle 2 (replicate 1)	8	0.3350	0.3941	0.3529	0.3309	0.4976
Yeast cell-cycle 2 (replicate 2)	8	0.3456	0.4536	0.4213	0.3624	0.2686
Yeast cell-cycle 3 (replicate 1)	10	0.2480	0.3222	0.3565	0.2690	0.4154
Yeast cell-cycle 3 (replicate 2)	10	0.2451	0.4301	0.4119	0.4048	0.2869
Yeast cell-cycle 4 (replicate 1)	19	0.1191	0.1361	0.1757	0.1217	0.1730
Yeast cell-cycle 4 (replicate 2)	19	0.1051	0.1427	0.1464	0.1097	0.1242
Yeast cell-cycle 5 (replicate 1)	28	0.1579	0.1943	0.1925	0.1627	0.1893
Yeast cell-cycle 5 (replicate 2)	28	0.1553	0.2083	0.2167	0.1719	0.2000

Network (dataset)	# Nodes	LEM (MCC)	TD-ARACNE (MCC)	Banjo DBN (MCC)		
In silico 1	3	1.0000	0.0000	-0.7906		
In silico 2	3	1.0000	0.0000	-0.7906		
In silico 6	3	1.0000	0.0000	0.5000		
In silico 7	3	1.0000	0.0000	0.5000		
In silico 3	5	0.6925	0.6999	-0.0965		
In silico 8	5	0.8018	0.4568	0.0364		
In silico 9	5	1.0000	0.6001	0.2041		
In silico 10	5	1.0000	0.5898	-0.2417		
In silico 4	10	0.7301	0.0935	0.0432		
In silico 11	10	0.6066	0.2572	-0.0050		
In silico 12	10	0.7115	0.2059	-0.0404		
In silico 13	10	0.5379	0.0948	0.0653		
In silico 14	10	0.6728	0.3091	0.0923		
In silico 15	10	0.6728	0.3393	0.0316		
In silico 16	10	1.0000	0.4419	0.2810		
In silico 17	10	1.0000	0.2703	0.0644		
In silico 18	10	0.5591	0.0961	0.1322		
In silico 19	10	0.3458	0.0576	-0.0151		
In silico 5	20	0.5784	0.3091	0.0124		
In silico 20	20	0.6895	0.3177	0.0925		
In silico 21	20	0.5114	0.1771	0.0436		
In silico 22	20	0.4483	0.0757	-0.0030		
Yeast cell-cycle 1 (replicate 1)	17	0.0062	0.0424	-0.0550		
Yeast cell-cycle 1 (replicate 2)	17	-0.0803	-0.0065	-0.0211		
Yeast cell-cycle 2 (replicate 1)	8	0.0778	0.0000	-0.1316		
Yeast cell-cycle 2 (replicate 2)	8	0.0000	0.1667	-0.2236		
Yeast cell-cycle 3 (replicate 1)	10	0.1555	0.0584	-0.0049		
Yeast cell-cycle 3 (replicate 2)	10	0.0737	0.1768	-0.1131		
Yeast cell-cycle 4 (replicate 1)	19	0.0048	0.1091	-0.0334		
Yeast cell-cycle 4 (replicate 2)	19	-0.0739	0.1080	-0.0048		
Yeast cell-cycle 5 (replicate 1)	28	-0.0211	0.0483	-0.0172		
Yeast cell-cycle 5 (replicate 2)	28	-0.0126	0.0338	-0.0389		
