# Supplementary material for: The Local Edge Machine: inference of dynamic models of gene regulation
Source: Genome Biol. 2016 Oct 19;17:214. doi: 10.1186/s13059-016-1076-z (PMC5072315; doi:10.1186/s13059-016-1076-z)
Supplement: Additional file 13 — Table: AUC-ROC scores for LEM on in silico networks with observational noise and prior information. As described in Additional file 1: Section 6, we added truncated Gaussian noise to the expression of each gene individually. The column labeled “Noise Level” indicates the variance of the noise as a percentage of the variance of the gene expression. We report the AUC-ROC score for LEM for five in silico networks with increasing access to prior information. (PDF 39 kb) [file 13059_2016_1076_MOESM13_ESM.pdf]

In silico 1	Has 3 priors						
Noise Level	No Prior	1/6 Prior	1/3 Prior	1/2 Prior	2/3 Prior	5/6 Prior	FullPrior
0	1	1	1	1	1	1	1
1	1	1	1	1	1	1	1
2	1	1	1	1	1	1	1
4	1	1	1	1	1	1	1
8	1	1	1	1	1	1	1
16	1	1	1	1	1	1	1
32	1	1	1	1	1	1	1
In silico 3	Has 5 priors						
Noise Level	No Prior	1/6 Prior	1/3 Prior	1/2 Prior	2/3 Prior	5/6 Prior	FullPrior
0	0.9900	0.9917	0.9941	0.9942	0.9966	0.9982	1
1	0.9966	0.9973	0.9977	0.9983	0.9990	0.9994	1
2	0.9900	0.9917	0.9922	0.9952	0.9971	0.9978	1
4	0.9933	0.9942	0.9953	0.9969	0.9982	0.9988	1
8	0.9900	0.9915	0.9937	0.9949	0.9967	0.9988	1
16	0.9867	0.9881	0.9909	0.9937	0.9957	0.9980	1
32	0.9933	0.9946	0.9956	0.9964	0.9978	0.9986	1
In silico 4	Has 14 priors						
Noise Level	No Prior	1/6 Prior	1/3 Prior	1/2 Prior	2/3 Prior	5/6 Prior	FullPrior
0	0.8884	0.8989	0.9102	0.9199	0.9304	0.9399	0.9504
1	0.8880	0.8998	0.9109	0.9218	0.9315	0.9406	0.9516
2	0.8861	0.8969	0.9076	0.9201	0.9312	0.9404	0.9512
4	0.8913	0.9015	0.9098	0.9203	0.9302	0.9397	0.9504
8	0.8365	0.8541	0.8699	0.8872	0.9047	0.9228	0.9372
16	0.8527	0.8684	0.8864	0.8988	0.9159	0.9305	0.9433
32	0.8258	0.8407	0.8585	0.8732	0.8862	0.9025	0.9172
In silico 9	Has 5 priors						
Noise Level	No Prior	1/6 Prior	1/3 Prior	1/2 Prior	2/3 Prior	5/6 Prior	FullPrior
0	1	1	1	1	1	1	1
1	1	1	1	1	1	1	1
2	1	1	1	1	1	1	1
4	1	1	1	1	1	1	1
8	0.9866	0.9915	0.9945	0.9958	0.9978	0.9990	1
16	0.9600	0.9726	0.9786	0.9883	0.9936	0.9979	1
32	0.9288	0.9457	0.9555	0.9709	0.9792	0.9872	0.9955

In silico 19	**Has 5 priors**						
**Noise Level**	**No Prior**	**1/6 Prior**	**1/3 Prior**	**1/2 Prior**	**2/3 Prior**	**5/6 Prior**	**FullPrior**
0	0.8824	0.8943	0.9038	0.9168	0.9241	0.9357	0.9439
1	0.8801	0.8922	0.9049	0.9144	0.9235	0.9344	0.9441
2	0.8911	0.8999	0.9094	0.9184	0.9273	0.9350	0.9425
4	0.8901	0.9002	0.9108	0.9192	0.9291	0.9376	0.9445
8	0.8815	0.8911	0.9002	0.9090	0.9183	0.9280	0.9366
16	0.8475	0.8574	0.8661	0.8724	0.8817	0.8922	0.9005
32	0.8406	0.8507	0.8595	0.8683	0.8794	0.8888	0.8974
