# Supplementary material for: The Local Edge Machine: inference of dynamic models of gene regulation
Source: Genome Biol. 2016 Oct 19;17:214. doi: 10.1186/s13059-016-1076-z (PMC5072315; doi:10.1186/s13059-016-1076-z)
Supplement: Additional file 14 — Table: Comparison of LEM with priors to existing algorithms on in vivo datasets. In this comparison, each algorithm has access to additional prior information, in the form of a known identity for each node (activator, repressor, neither, or both/unknown). See Additional file 1: Section 11 for a description of the yeast cell-cycle networks used. (PDF 35 kb) [file 13059_2016_1076_MOESM14_ESM.pdf]

Network (dataset)	# Nodes	LEM (AUC)	Inferelator (AUC)	Granger Causality (AUC)	Hill-DBN (AUC)	Jump3 (AUC)
Yeast cell-cycle 1 (replicate 1)	17	0.9889	0.7710	0.9121	0.9129	0.8740
Yeast cell-cycle 1 (replicate 2)	17	0.9851	0.8201	0.9177	0.8874	0.7978
Yeast cell-cycle 2 (replicate 1)	8	0.9682	0.7670	0.7955	0.7391	0.7645
Yeast cell-cycle 2 (replicate 2)	8	0.9626	0.8047	0.8161	0.7849	0.7486
Yeast cell-cycle 3 (replicate 1)	10	0.8814	0.7236	0.8198	0.7666	0.7797
Yeast cell-cycle 3 (replicate 2)	10	0.8779	0.7586	0.8035	0.8349	0.7313
Yeast cell-cycle 4 (replicate 1)	19	0.9545	0.6912	0.9060	0.8997	0.8277
Yeast cell-cycle 4 (replicate 2)	19	0.9466	0.7723	0.9076	0.9007	0.7597
Yeast cell-cycle 5 (replicate 1)	28	0.8235	0.5993	0.8293	0.8054	0.7166
Yeast cell-cycle 5 (replicate 2)	28	0.8166	0.6436	0.8506	0.8165	0.7221
**Network (dataset)**	**# Nodes**	**LEM (AUPR)**	**Inferelator (AUPR)**	**GrangerCausality (AUPR)**	**Hill-DBN (AUPR)**	**Jump3 (AUPR)**
Yeast cell-cycle 1 (replicate 1)	17	0.6749	0.3251	0.2869	0.2637	0.2666
Yeast cell-cycle 1 (replicate 2)	17	0.5749	0.3105	0.3170	0.2197	0.3157
Yeast cell-cycle 2 (replicate 1)	8	0.7649	0.3617	0.3250	0.3028	0.4571
Yeast cell-cycle 2 (replicate 2)	8	0.6513	0.4267	0.4049	0.3319	0.2333
Yeast cell-cycle 3 (replicate 1)	10	0.4488	0.2999	0.3500	0.2628	0.3843
Yeast cell-cycle 3 (replicate 2)	10	0.4194	0.4035	0.4032	0.3890	0.2665
Yeast cell-cycle 4 (replicate 1)	19	0.4900	0.2233	0.3093	0.2321	0.2488
Yeast cell-cycle 4 (replicate 2)	19	0.4109	0.2592	0.3040	0.2416	0.2376
Yeast cell-cycle 5 (replicate 1)	28	0.2888	0.2174	0.2561	0.2294	0.2175
Yeast cell-cycle 5 (replicate 2)	28	0.2456	0.2504	0.2959	0.2481	0.2466
**Network (dataset)**	**# Nodes**	**LEM (MCC)**	**TD-ARACNE (MCC)**	**Banjo (MCC)**		
Yeast cell-cycle 1 (replicate 1)	17	0.7379	0.1375	0.0676		
Yeast cell-cycle 1 (replicate 2)	17	0.5438	0.0626	0.1102		
Yeast cell-cycle 2 (replicate 1)	8	0.6831	0.0808	0.0605		
Yeast cell-cycle 2 (replicate 2)	8	0.5855	0.2857	-0.0286		
Yeast cell-cycle 3 (replicate 1)	10	0.4453	0.1295	0.1237		
Yeast cell-cycle 3 (replicate 2)	10	0.4453	0.2597	0.0336		
Yeast cell-cycle 4 (replicate 1)	19	0.5761	0.1950	0.0908		
Yeast cell-cycle 4 (replicate 2)	19	0.4406	0.2649	0.1079		
Yeast cell-cycle 5 (replicate 1)	28	0.1845	0.1003	0.0775		
Yeast cell-cycle 5 (replicate 2)	28	0.1438	0.1289	0.0486		
