# Supplementary material for: The Local Edge Machine: inference of dynamic models of gene regulation
Source: Genome Biol. 2016 Oct 19;17:214. doi: 10.1186/s13059-016-1076-z (PMC5072315; doi:10.1186/s13059-016-1076-z)
Supplement: Additional file 15 — Table: AUC-ROC scores for LEM on yeast cell-cycle networks with partial access to prior information. For each of the yeast cell-cycle networks, we collected the known identities of the nodes: activator, repressor, both/unknown, or neither (see Additional file 2). The number of such pieces of information for each network is presented in the column labeled “# Priors.” The other columns present the average AUC-ROC score obtained by LEM on each network after randomly selecting the indicated fraction of the possible pieces of prior information as input to LEM 100 times (except that no random selections are necessary in the columns denoted “NoPrior” and “FullPrior”). (PDF 30 kb) [file 13059_2016_1076_MOESM15_ESM.pdf]

Network (dataset)	# Priors	No Prior	1/6 Prior	1/3 Prior	1/2 Prior	2/3 Prior	5/6 Prior	Full Prior
Yeast cell-cycle 1 (replicate 1)	25	0.8693	0.8888	0.9108	0.9331	0.9519	0.9733	0.9889
Yeast cell-cycle 1 (replicate 2)	25	0.8465	0.8695	0.8927	0.9163	0.9402	0.9629	0.9854
Yeast cell-cycle 2 (replicate 1)	7	0.8460	0.8693	0.8902	0.9100	0.9339	0.9504	0.9682
Yeast cell-cycle 2 (replicate 2)	7	0.8404	0.8646	0.8841	0.9055	0.9260	0.9441	0.9626
Yeast cell-cycle 3 (replicate 1)	9	0.7092	0.7388	0.7662	0.7985	0.8264	0.8534	0.8832
Yeast cell-cycle 3 (replicate 2)	9	0.6957	0.7246	0.7580	0.7866	0.8181	0.8487	0.8776
Yeast cell-cycle 4 (replicate 1)	27	0.7409	0.7771	0.8120	0.8480	0.8843	0.9204	0.9544
Yeast cell-cycle 4 (replicate 2)	27	0.7208	0.7589	0.7959	0.8334	0.8724	0.9101	0.9464
Yeast cell-cycle 5 (replicate 1)	32	0.5138	0.5664	0.6179	0.6693	0.7196	0.7724	0.8236
Yeast cell-cycle 5 (replicate 2)	32	0.4804	0.5369	0.5915	0.6486	0.7036	0.7596	0.8166
