# Supplementary material for: The Local Edge Machine: inference of dynamic models of gene regulation
Source: Genome Biol. 2016 Oct 19;17:214. doi: 10.1186/s13059-016-1076-z (PMC5072315; doi:10.1186/s13059-016-1076-z)
Supplement: Additional file 18 — Table: Circadian phenotypes of target genes screened by RNAi in NIH3T3 Per2:Luc circadian bioluminescence reporter cells. The p values for cycling were calculated using a wavelet-based method [53]. The significance of period length changes was determined using a two-way t-test. “AR” is an abbreviation of “arrhythmic.” A p-value cutoff of 0.05 was used to determine whether the period length change from Neg si treatment was statistically significant. Five out of ten target genes were found to have a significant circadian phenotype. (PDF 36 kb) [file 13059_2016_1076_MOESM18_ESM.pdf]

Gene Name	Period Change (h)	P-value	Individual siRNAs with phenotype
**Controls**			
Bmal1	AR		4/4
Cry1	AR		4/4
Cry2	+3.95 ± 0.46	<0.001	4/4
**Targets**			
Ankrd23	AR		3/4
Cml5	+1.19 ± 0.09	0.010	4/4
Fus	-0.36 ± 0.66	0.148	
Gnl3	-0.35 ± 0.18	0.141	
Mgrn1	+0.66 ± 0.49	0.110	
Nup62	+1.76 ± 0.44	0.004	3/4
P2ry1	+1.37 ± 0.21	0.027	1/2
Peg3	+0.56 ± 0.43	0.128	
Tsc22d2	+1.72 ± 0.31	0.002	2/2
Tsc22d3	+0.32 ± 0.19	0.177	
