# Supplementary material for: The Local Edge Machine: inference of dynamic models of gene regulation
Source: Genome Biol. 2016 Oct 19;17:214. doi: 10.1186/s13059-016-1076-z (PMC5072315; doi:10.1186/s13059-016-1076-z)
Supplement: Additional file 19 — Table: Multiple hypothesis corrections for analysis of circadian target genes. The column “BHq val” contains the Benjamini–Hochberg corrected q values [54]. Four out of ten candidate genes remain significant after multiple hypothesis correction. (PDF 30 kb) [file 13059_2016_1076_MOESM19_ESM.pdf]

	**p-val**	**BHq val**
Neg.si	1.000	1.000
Ankrd23	NA	NA
Cml5	0.010	0.033
Fus	0.148	0.230
Gnl3	0.172	0.230
Mgrn1	0.110	0.230
Nup62	0.004	0.019
P2ry1	0.027	0.071
Peg3	0.128	0.230
Tsc22d2	0.002	0.013
Tsc22d3	0.177	0.230
Cry2	0.000	0.003

**Arryhthmic (AR)
