# Supplementary material for: The Local Edge Machine: inference of dynamic models of gene regulation
Source: Genome Biol. 2016 Oct 19;17:214. doi: 10.1186/s13059-016-1076-z (PMC5072315; doi:10.1186/s13059-016-1076-z)
Supplement: Additional file 24 — Table: Comparison of methods using AUC-ROC and AUPR scores for the 100-node networks. Scores are reported for the networks in silico 23 and in silico 24 on both the signed and unsigned edge inference challenges. (PDF 31 kb) [file 13059_2016_1076_MOESM24_ESM.pdf]

Signed edge inference task						
**Network**	**# Nodes**	**LEM (AUC)**	**Inferelator (AUC)**	**Granger Causality (AUC)**	**Hill-DBN (AUC)**	**Jump3 (AUC)**
In silico 23	100	1.0000	0.9878	0.9924	0.9874	0.9975
In silico 24	100	0.9707	0.8250	0.8396	0.7950	0.5896
**Signed edge inference task**						
**Network**	**# Nodes**	**LEM (AUPR)**	**Inferelator (AUPR)**	**Granger Causality (AUPR)**	**Hill-DBN (AUPR)**	**Jump3 (AUPR)**
In silico 23	100	0.5049	0.1361	0.1271	0.2426	0.4974
In silico 24	100	0.2710	0.0467	0.0287	0.0301	0.0142
**Unsigned edge inference task**						
**Network**	**# Nodes**	**LEM (AUC)**	**Inferelator (AUC)**	**Granger Causality (AUC)**	**Hill-DBN (AUC)**	**Jump3 (AUC)**
In silico 23	100	1.0000	0.9903	0.9949	0.9898	1.0000
In silico 24	100	0.9422	0.8266	0.8414	0.7964	0.5901
**Unsigned edge inference task**						
**Network**	**# Nodes**	**LEM (AUPR)**	**Inferelator (AUPR)**	**Granger Causality (AUPR)**	**Hill-DBN (AUPR)**	**Jump3 (AUPR)**
In silico 23	100	0.5049	0.2723	0.2542	0.4852	0.9948
In silico 24	100	0.2711	0.0934	0.0574	0.0602	0.0285
