# Supplementary material for: The Local Edge Machine: inference of dynamic models of gene regulation
Source: Genome Biol. 2016 Oct 19;17:214. doi: 10.1186/s13059-016-1076-z (PMC5072315; doi:10.1186/s13059-016-1076-z)
Supplement: Additional file 25 — Table: Comparison of methods using MCC scores for the 100-node networks. Scores are reported for the networks in silico 23 and in silico 24 on both the signed and unsigned edge inference challenges. (PDF 29 kb) [file 13059_2016_1076_MOESM25_ESM.pdf]

Signed edge inference task				
**Network**	**# Nodes**	**LEM (MCC)**	**TD-ARACNE (MCC)**	**Banjo DBN (MCC)**
In silico 23	100	0.9950	0.0001	-0.0072
In silico 24	100	0.3930	0.0750	0.0308
**Unsigned edge inference task**				
**Network**	**# Nodes**	**LEM (MCC)**	**TD-ARACNE (MCC)**	**Banjo DBN (MCC)**
In silico 23	100	0.9949	0.0002	-0.0102
In silico 24	100	0.3899	0.1064	0.0437
