# Supplementary material for: The Local Edge Machine: inference of dynamic models of gene regulation
Source: Genome Biol. 2016 Oct 19;17:214. doi: 10.1186/s13059-016-1076-z (PMC5072315; doi:10.1186/s13059-016-1076-z)
Supplement: Additional file 26 — Table: Run times of LEM and the method of Mazur et al. Run times are reported for LEM and the method of Mazur et al. [48] on four in silico networks, each with three nodes. The method of Mazur et al. was run with all default settings, including 10,000 steps of burn-in and 50,000 steps of iteration for the MCMC computations. Although LEM is parallelizable, here we report the amount of time LEM would take to run on a single core. (PDF 34 kb) [file 13059_2016_1076_MOESM26_ESM.pdf]

Network	# Nodes	Mazur *et al.* run time (secs)	LEM run time (secs)
In silico 1	3	38813.20	402.25
In silico 2	3	35610.86	505.87
In silico 6	3	38635.03	456.09
In silico 7	3	37566.28	333.58
